# Structure and Ionic Conductivity of Halide Solid Electrolytes Based on NaAlCl_4_ and Na_2_ZnCl_4_

**DOI:** 10.1002/advs.202507224

**Published:** 2025-05-28

**Authors:** Hao Guo, Michael Häfner, Helen Grüninger, Matteo Bianchini

**Affiliations:** ^1^ Department of Biology, Chemistry and Earth Sciences University of Bayreuth Universitätstraße 30 95447 Bayreuth Germany; ^2^ Bavarian Center for Battery Technology (BayBatt) Weiherstraße 26 95448 Bayreuth Germany

**Keywords:** sodium, solid‐electrolyte, XRD, NMR, MD

## Abstract

Sodium‐based solid‐state batteries may represent safe and cost‐effective energy storage devices, complementing Li‐ion for specific applications such as grid storage. Thus, sustainable solid‐state electrolytes (SSE) with high ionic conductivity need to be developed. Sodium metal halide SSEs have attracted significant attention due to their ionic conductivity, electrochemical stability, and adequate processibility. Here, SSE based on NaAlCl_4_ (NAC) and Na_2_ZnCl_4_ (NZC) are investigated, nominally Na_1+*x*_Zn_*x*_Al_1−*x*_Cl₄. Compounds synthesized by ball‐milling and investigated by X‐ray diffraction revealed a two‐phase system, with a solid solution in the Na_2_ZnCl_4_‐type structure extending to ≈34(3)% Al substitution. EIS results demonstrate the highest ionic conductivity is near the miscibility gap edge *(x* = 0.625), where σ is increased by several orders of magnitude as compared to NZC and reaches 1.5×10⁻^5^ S cm^−1^ at 25 °C, above the values of Na_2_ZnCl_4_/NaAlCl_4_. The combined use of molecular dynamics simulations and nuclear magnetic resonance distinctly elucidates the importance of achieving enough Na⁺ vacancies in both Na sublattices in NZC‐type structures. This work introduces a novel class of SSE based on the NZC olivine structure, demonstrates that they can be used as catholytes to assemble working solid‐state sodium batteries, and provides insights into the correlation between composition, crystalline structure, and ionic conduction pathways.

## Graphical abstract

A solid solution forms in the Na_2_ZnCl_4_‐type phase when Al³⁺ substitutes in the Zn²⁺ tetrahedral site; correspondingly, Na^+^ vacancies form in the Na1 site, allowing for Na hopping. The highest ionic conductivity reaches 1.5*10^−5^ S cm^−1^ at 25 °C at the edge of the miscibility gap. The family of materials and their increased conductivity was investigated in detail by x‐ray diffraction, nuclear magnetic resonance and, and molecular dynamics simulations.

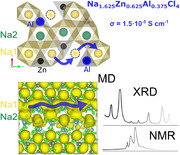

## 1 Introduction

Lithium‐ion batteries (LIBs) have been successfully applied in electric vehicles, portable devices, and energy storage solutions.^[^ ^1^, ^2^, ^3^ ^]^ However, safety concerns, the scarcity of lithium resources, and the resulting fluctuating prices remain the Achilles' heel of LIBs.^[^ ^4^ ^]^ Sodium solid‐state batteries (SSBs) have attracted enormous attention and are regarded as a potential alternative to LIBs. Ideally, sodium SSBs offer reduced costs due to the abundance of sodium on earth and potential improved safety because of the use of inflammable solid‐state components.^[^ ^5^ ^]^ One of the perspectives of sodium SSBs is applying them in large‐scale energy storage systems owing to their excellent cost‐effectiveness and similar manufacturing process to LIBs.^[^ ^6^, ^7^ ^]^ The key component that significantly impacts solid‐state batteries' properties and performance is the solid‐state electrolyte. The design of SSEs must balance various properties, such as superionic conductivities (>10^−3^ S cm^−1^), electrochemical stability against both electrodes, and processability.^[^ ^8^, ^9^, ^10^ ^]^ Traditional inorganic Na‐based SSE families include oxide ceramics (NASICON‐type oxides,^[^ ^11^, ^12^ ^]^ β‐Al_2_O_3_^[^ ^13^ ^]^) and sulfides (Na_3_PS_4_,^[^ ^14^ ^]^ Na_2.9_Sb_0.9_W_0.1_S_4_,^[^ ^15^, ^16^ ^]^ Na_11_Sn_2_PS_12_‐type^[^ ^17^, ^18^ ^]^). However, both have drawbacks: oxide ceramics have high interfacial impedance and require high sintering temperatures. Meanwhile, sulfides exhibit a narrow electrochemical stability window, especially the incompatibility with high‐voltage cathodes and sodium metal.^[^ ^19^ ^]^

Sodium metal halides have emerged as promising catholyte materials, offering excellent mechanical processability (via cold‐pressing) and compatibility with high‐voltage electrodes. In 2018, Asano et al. demonstrated the feasibility of using ball‐milling to create a disordered cation (Y^3+^) arrangement in the Li_3_YCl_6_ (LYC) crystal. This method boosted the ionic conductivity of LYC to ≈0.5×10^−3^ S cm^−1^ by opening Li^+^ transport pathways through disordering and stacking faults.^[^ ^20^ ^]^ Na‐based halide SSEs have also been reported and can be divided into different families according to cation valence: NaM^5+^Cl_6_ (M = Nb, Ta),^[^ ^21^, ^22^ ^]^ Na_2_Zr^4+^Cl_6_,^[^ ^23^ ^]^ Na_3_M^3+^Cl_6_ (M = In, Y, Er, Sc, Sm),^[^ ^22^, ^24^, ^25^, ^26^ ^]^ NaAl^3+^Cl_4_.^[^ ^27^ ^]^ Most of them exhibit insufficient ionic conductivity of the order of 10^−6^ S/cm. Tuning the concentration of vacancies via aliovalent substitution is a viable strategy to address this issue. The targeted introduction of vacancies via the mixing of two cations with different valences is widely employed to directly influence the ionic conductivity of the compound.^[^ ^22^, ^24^, ^28^, ^29^ ^]^ Examples include Na_3‐_ *_x_* Y_1‐_ *_x_* Zr*_x_* Cl_6_^[^ ^30^, ^31^, ^32^ ^]^ and Na_3−_ *_x_* In_1−_ *_x_* Zr*_x_* Cl_6_.,^[^ ^33^, ^34^ ^]^ reaching ionic conductivities around 6.6 × 10^−5^ S cm^−1^,^[^ ^30^ ^]^ which is normally 1–3 orders of magnitude larger than that of the associated end members. Recently, the study of amorphous sodium oxychloride phases as SSEs (NaMOCl_4_, M = Nb, Ta) has drawn enormous attention due to their boost in ionic conductivity (≈10^−3^ S cm^−1^), while maintaining high oxidation stability.^[^ ^35^, ^36^, ^37^, ^38^, ^39^, ^40^, ^41^ ^]^Lin et al. reported a dual‐anion sodium superionic glass 0.5Na_2_O_2_‐TaCl_5_ with a high ionic conductivity of 4.6 × 10^−3^ S cm^−1^ at 25 °C.^[^ ^39^ ^]^ The high value is mainly attributed to the incorporation of oxygen, which forms Ta‐centered [TaO*_x_* Cl*_y_*] units and oligomeric Ta‐centered polyhedra which connect via corner‐shared oxygen. While most amorphous sodium (oxy)chloride SSEs to date are based on rare earth metals, especially Ta and Nb, limiting their economic potential, some investigations based on NaAlCl_4_ and anion‐doped NaAlCl_4‐2_ *_x_* O*_x_* have been recently reported.^[^ ^35^, ^42^, ^43^ ^]^ NaAlCl_4_ is known for its past use (in the molten state) as a catholyte in ZEBRA batteries.^[^ ^44^ ^]^ This material may offer a cost‐effective alternative due to its raw materials' abundance and low cost. However, its conductivity as a solid is still low. Nonetheless, its usage as catholyte (but not as a separator) in SSBs was recently demonstrated.^[^ ^27^ ^]^ The recent modifications of NaAlCl_4_ further improved its conductivity but significantly amorphized it.^[^ ^42^, ^43^ ^]^ In this work, we aim to increase the conductivity of crystalline NaAlCl_4_ without using rare earth elements. We synthesized and studied the influence of Zn^2+^ aliovalent substitution in NaAlCl_4_ (according to the nominal Na_1+_ *_x_* Zn*_x_* Al_1‐_ *_x_* Cl_4_ formula unit). Since the second end member, Na_2_ZnCl_4,_ belongs to a different structural type than NaAlCl_4_, a solid solution is also not guaranteed. Therefore, we also investigated the introduction of Al^3+^ (and Na vacancies) in the Na_2_ZnCl_4_ end member. The whole series of samples has been synthesized by mechanochemical methods. Their structure and properties will be discussed based on X‐ray diffraction (XRD), synchrotron X‐ray diffraction (sXRD), electrochemical impedance spectroscopy (EIS), and solid‐state nuclear magnetic resonance (ss‐NMR) spectroscopy. Ss‐NMR spectroscopy was conducted to investigate the local environment of Na and explore the origin of the conductivity increase, in combination with computational methods such as density functional theory (DFT) and molecular dynamics (MD) simulations.

## 2 Results and Discussion

### 2.1 Structure and Conductivity of NaAlCl_4_

NaAlCl_4_ has been applied as a catholyte in ZEBRA Batteries due to its Na^+^ ionic conductivity in the molten state.^[^ ^44^ ^]^ As a solid, NaAlCl_4_ (NAC) adopts an orthorhombic space group (*P*2_1_2_1_2_1_) at room temperature. Mechanochemically prepared NAC with such a crystal structure exhibits a Na⁺ conductivity of 3.9 × 10⁻⁶ S cm^−1^ at 30 °C. According to the work of Jung et al.,^[^ ^27^ ^]^ ball‐milled NAC contains two crystallographic Na positions with occupancies of Na1 = 0.673 and Na2 = 0.327. However, first‐principles calculations from previous work revealed that the structural model with all Na⁺ ions occupying the Na1 site is energetically more favorable than the model where Na⁺ ions occupy both Na1 and Na2 sites, with only a minimum difference of 5 meV/atom.^[^ ^27^ ^]^

**Figure 1:**
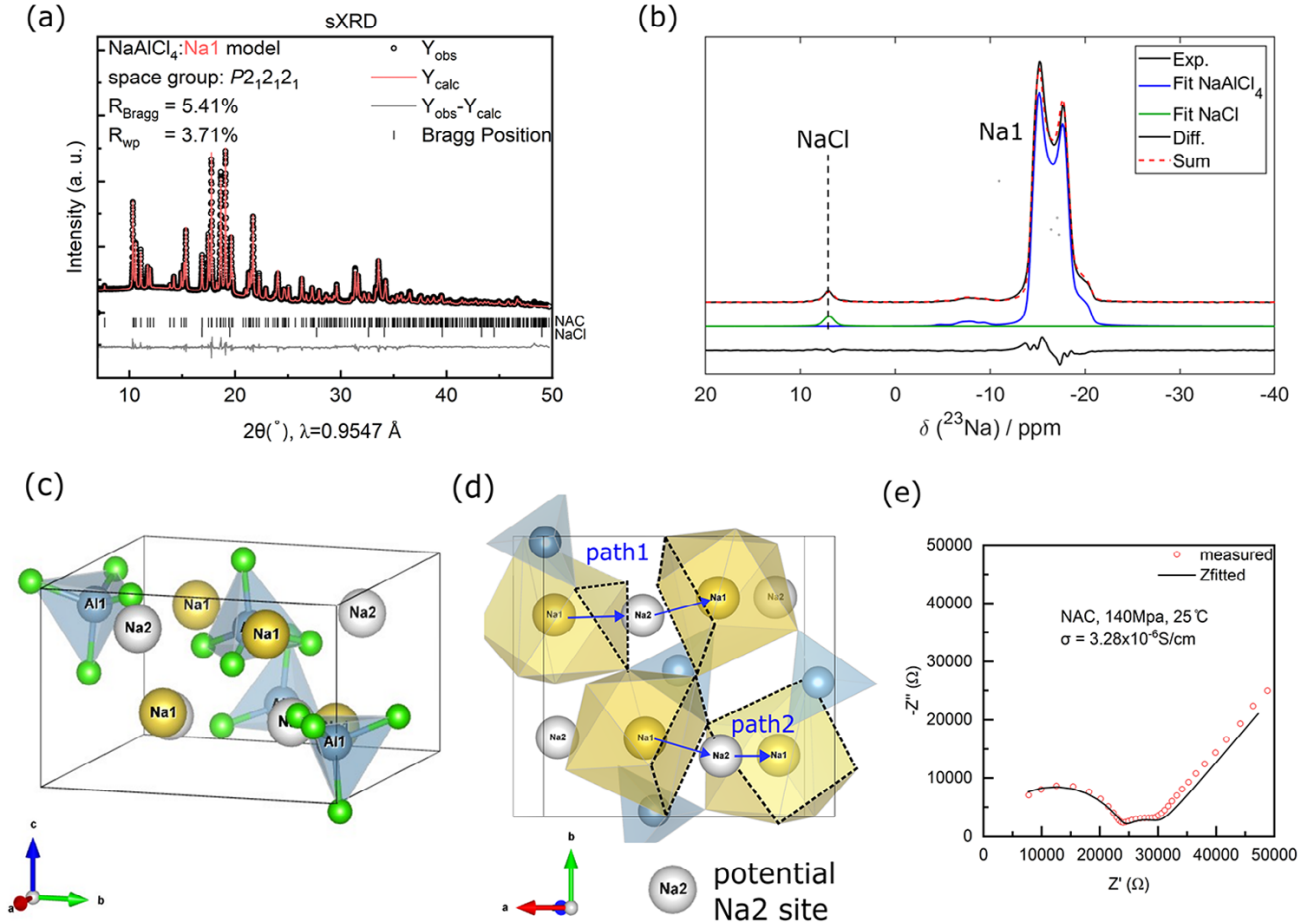
a) Synchrotron X‐ray diffraction pattern of NAC and related Rietveld refinement. b) ^23^Na MAS NMR spectrum displaying the central transition of NAC, which can be attributed to a single Na environment. A minor signal related to NaCl impurity is also observable. c) Crystal structure of NaAlCl_4_. Schematic illustration of Na1 (yellow spheres) and potential Na2 (grey spheres) positions, which are shown as vacancies. Blue tetrahedra are AlCl_4_^−^. d) Crystal structure of NAC schematically shows two types of Na^+^ migration pathways. Cl^−^ ions are not shown. e) Nyquist plot and corresponding fitting of ball‐milled NAC sample. EIS was measured at 25 °C under an external pressure of 140 MPa.

We synthesized NaAlCl_4_ mechanochemically and verified its crystal structure via XRD. The measured pattern is gathered in Figure S1 (Supporting Information). Despite the synthesis route, the material appears well crystallized, and the reflections can be well indexed with the *P*2_1_2_1_2_1_ space group, with merely additional peaks from unreacted NaCl impurities below 1 wt.%. The presence of minor NaCl in ball‐milled sodium halide SSEs was also found in previous work but was identified as non‐detrimental to ionic conduction.^[^ ^27^, ^31^ ^]^ The crystal structure is based on Al^3^⁺ occupying the Wyckoff *4a* site, forming AlCl₄⁻ tetrahedra due to its small ionic radius (53.5 pm). The XRD patterns can be refined in two ways: using either a single sodium site (Na1 model) or with two sodium sites (the Na1+2 model). Figure S1 and Table S1 (Supporting Information) show the Rietveld refinement of these two structural models. The Rietveld refinement results based on both models show no significant differences in terms of R_Bragg_ and R_wp_. However, the refinement results for the Na1+2 model (Figure S1b, Supporting Information) demonstrate that the Na2 occupancy in the Na1+2 model is effectively zero within error, suggesting that Na⁺ ions strongly prefer not to occupy the Na2 sites. To further verify the potential occupancy of the Na2 site, the same NAC was measured via synchrotron XRD with high angular resolution and counting statistics. **Figure** **1**a depicts the refinement results (details are listed in Table S2, Supporting Information). Based on the calculated and observed XRD pattern, difference Fourier maps were calculated to verify potential mismatches in the electronic density. Figure S2 (Supporting Information) demonstrates the positive (yellow) and negative (blue) electron density iso‐surfaces in the structure. When the existence of Na^+^ in Na2 is ignored in the refinement based on the Na1 model, a positive (yellow) iso‐surface shall appear. However, the Na2 sites are not adjacent to any electron density iso‐surface, which implies no potential occupation of Na^+^ in the Na2 site.

Additionally, ^2^ ^3^Na ss‐NMR spectroscopy was performed to determine the distribution of Na local environments in Na SSEs.^[^ ^45^ ^]^ Figure 1b displays the ^2^ ^3^Na magic angle spinning (MAS) NMR spectrum of NAC at room temperature, where the NaCl peak at 7.0 ppm is observable. Notably, the ^23^Na signal at δ_iso_ = −14.2 ppm is attributed to a single type of Na environment with a quadrupolar line shape (C_Q_ = 1.1 MHz; η = 0.05). The small signals from −5 to −10 ppm stem from residual satellite transitions of the quadrupolar interaction, which are not fully suppressed by magic angle spinning. The ss‐NMR results confirm the reliability of the Rietveld refinement and corroborate the presence of only one Na environment in the crystal structure of NAC.

The AlCl₄⁻ tetrahedra are isolated from each other but are connected to Na⁺ capped‐trigonal prisms through both edge‐sharing and corner‐sharing (Figure 1c). All Na⁺ cations occupy the Wyckoff *4a* site, and the seven Cl⁻ anions surrounding one Na⁺ form a capped‐trigonal prism, which generates a framework through corner‐sharing. The facile Na⁺ ionic conduction pathway in NAC is illustrated in Figure 1d. Two different Na⁺ conduction mechanisms are displayed. Path 1 involves Na1 hopping through a triangular face and a rectangular face between two unconnected prisms to another Na1 site. Path 2 involves Na1 hopping through two rectangular faces between two corner‐sharing prisms. Both paths feature the Na2 sites as intermediate sites. To quantify the ionic conductivity, we measured EIS data. Figure 1e shows the Nyquist plot and Z‐fitting of the equivalent circuit (details provided in Figure S3, Supporting Information). The semicircle on the left side at high frequencies represents the Na⁺ transport processes in both the bulk and grain boundaries, which are difficult to deconvolute due to their similar capacitance values (≈10⁻⁹ F). The smaller semicircle on the right side at low frequencies with capacitance around 10⁻⁶ F, can be attributed to the transport mechanism at the SSE/hard metal interface.^[^ ^46^ ^]^ From the EIS‐fitting, we obtained a conductivity of 3.28 × 10^−6^ S cm^−1^, which is in good agreement with literature values.^[^ ^27^ ^]^ Therefore, it is suggested that the ionic conduction in NAC originates from two factors: i) the movement of AlCl₄^⁻^ tetrahedra, facilitated by their isolated nature, and ii) the empty “Na2” sites in the unit cell, which serve as intermediate sites for Na⁺ hopping from one site to another. To be noted that the unoccupied free volume in the unit cell is a prerequisite for efficient ionic conduction.^[^ ^29^, ^33^ ^]^

### 2.2 Structure and Conductivity of Na_2_ZnCl_4_

**Figure 2:**
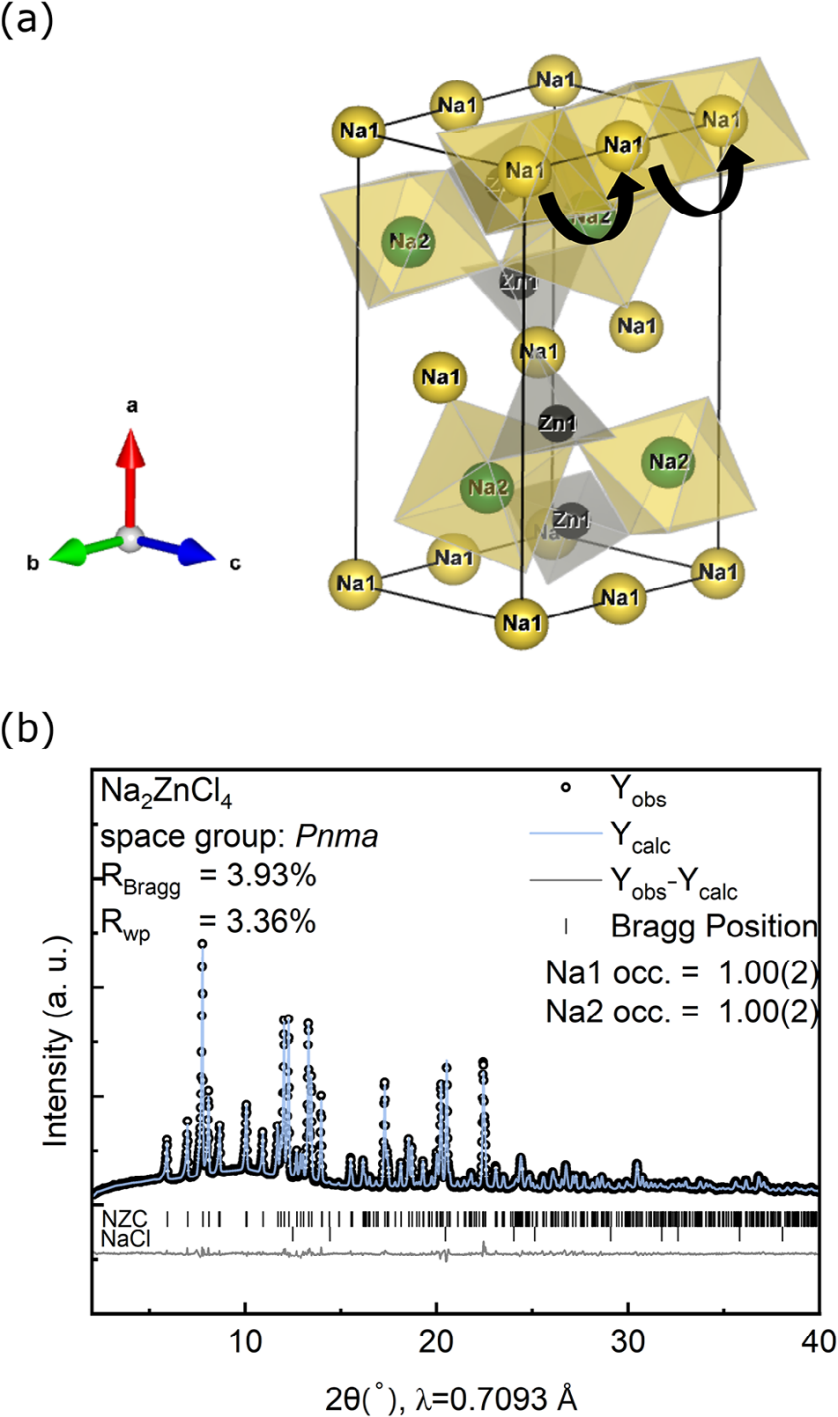
a) Crystal structure of Na_2_ZnCl_4_ (NZC) in space group *Pnma*. b) Rietveld refinement of the NZC structural model.

Na₂ZnCl₄ (NZC) crystallizes in the *Pnma* space group without exhibiting any polymorphic behavior. The structure of NZC is shown in **Figure** **2**a, as confirmed by XRD (Figure 2b). Interestingly, the olivine‐type structure is the same as the well‐studied LiFePO₄ (LFP) positive electrode material.^[^ ^47^, ^48^, ^49^, ^50^ ^]^ Like the metal cations Li^+^ and Fe^2+^ in LFP, in NZC the Na⁺ is coordinated by six Cl⁻ ions to form an octahedron. Na1 occupies the Wyckoff *4a* site located at the corner of the unit cell, corresponding to the Li⁺ site in the LFP structure. Na2 resides at the Wyckoff *4c* site, which corresponds to the Fe^2^⁺ site in LFP. Both Na1 and Na2 polyhedral form sublattices in the *bc*‐plane, stacking in an ABAB pattern (hexagonal close packing of the anions). The Na1 octahedra are connected through edge‐sharing, and the chain along the [010] direction is shown in Figure 2a. Furthermore, ZnCl₄^2^⁻ tetrahedra are located between these chains in the Na1 sublattice, connecting them by corner‐sharing. The Na2 sublayer is built exclusively of corner‐shared Na2 octahedra.

Perfect LFP crystals are known to possess high Li conductivity along the *b* direction.^[^ ^48^ ^]^ Likewise, the polyhedral network in the NZC structure provides a potential continuous Na⁺ conduction pathway, as indicated by black arrows in Figure 2a. A chain of Na1 octahedra on *4a* sites connects through face‐shared vacant tetrahedral sites, allowing Na⁺ to hop to neighboring octahedral sites along the [010] direction. However, due to the fully occupied Na1 sites in NZC, no free volume is available for Na⁺ conduction. The Na^+^ ionic conductivity in NZC is below 10^−10^ S/cm, depicted in Figure S4 (Supporting Information). However, one could wonder how the creation of vacancies along the Na channels could affect the conductivity, which we will show next.

### 2.3 Structure of the Potential Solid Solution Na_1+_ *_x_* Zn*_x_* Al_1‐_ *_x_* Cl_4_

**Figure 3:**
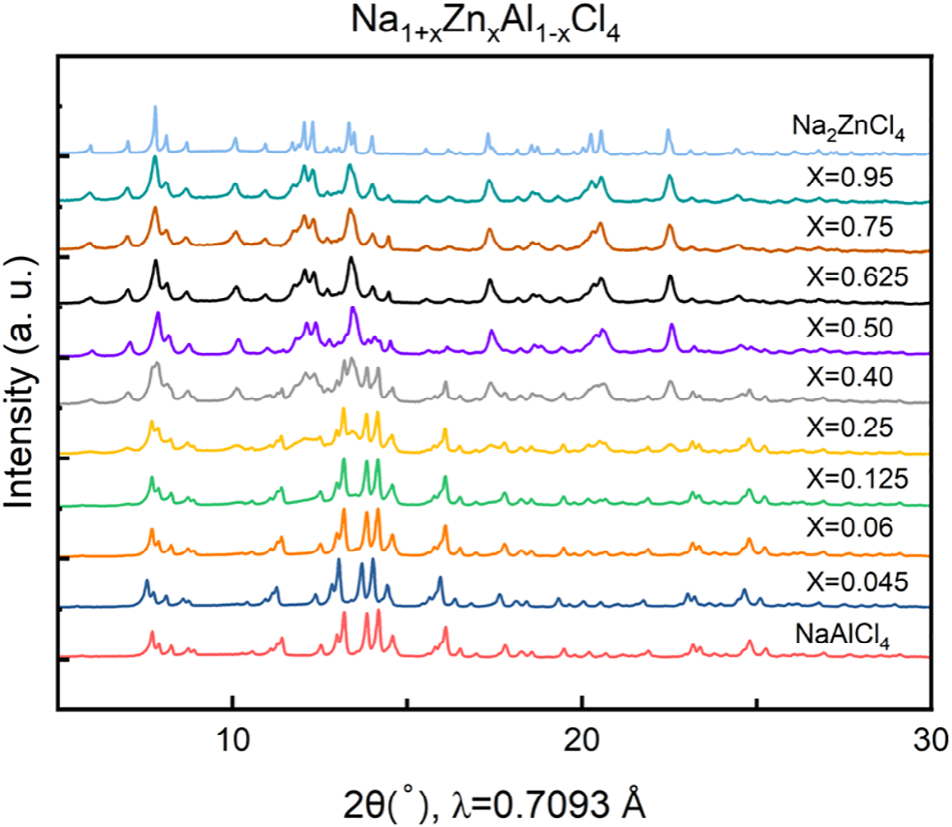
X‐ray diffraction patterns of Na_1+_ *_x_* Zn*_x_* Al_1‐_ *_x_* Cl_4_ (0 ≤ *x* ≤ 1).

In our recent work, we demonstrated the potential stability and synthesizability of mixed compounds along the solid solution Na_1+_ *_x_* Zn*_x_* Al_1‐_ *_x_* Cl_4_.^[^ ^51^ ^]^ Here we verify experimentally the existence of some of these compounds. Indeed, we studied the influence of aliovalent substitution of Zn^2+^ and Al^3+^ in the two end members. A series of Na_1+_ *_x_* Zn*_x_* Al_1‐_ *_x_* Cl_4_ (NZAC) samples were mechanochemically synthesized analogously to NAC and NZC. The XRD patterns collected for nominal compositions Na_1+_ *_x_* Zn*_x_* Al_1‐_ *_x_* Cl_4_ (0 ≤ *x* ≤ 1) are shown in **Figure** **3**. All reflections can be attributed to either NAC or NZC, indicating that no additional phases were formed as the substitution degree (*x*) varied. On the other hand, some peaks shift appreciably for compositions close to the NZC end member. Rietveld refinements of the X‐ray diffraction patterns were conducted to obtain detailed structural information. All refinement parameters and constraints are provided in the Supporting Information Tables S1–S13 (Supporting Information).

**Figure 4:**
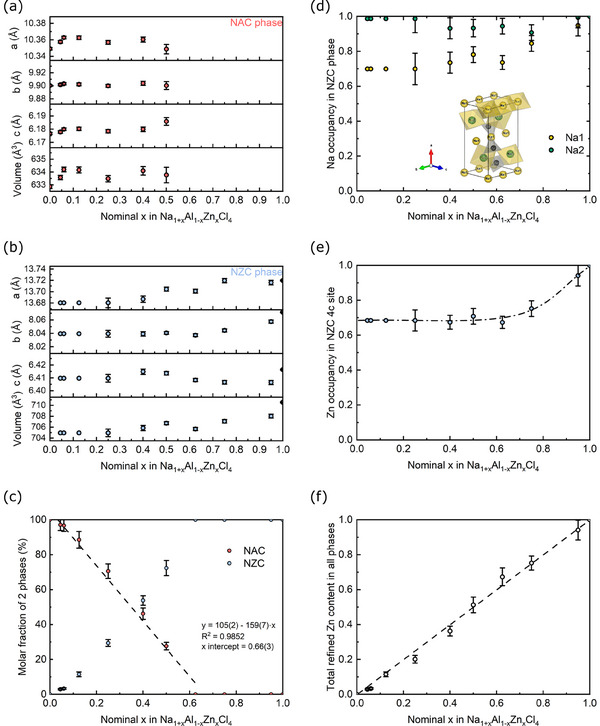
Rietveld refinement results for structural models of NZAC compositions. The nominal Zn^2+^ content is plotted against a) lattice parameters in the NAC‐type phase, b) lattice parameters in the NZC‐type phase, c) molar phase fraction of NAC and NZC, with linear interpolation, d) Na^+^ occupancies at Wyckoff *4a* and *4c* sites in NZC phase, e) Zn^2+^ occupancies at Wyckoff *4c* sites, f) total refined Zn content in all phases.

**Figure** **4** gathers all refinement results, which were conducted with a two‐phase model containing both NAC and NZC if the reflections of both were present in the diffractogram. Figure 4a,b show the evolution of unit cell parameters in both the NAC‐related and NZC‐related end‐member structures. Initially, a small increase in the unit cell parameters of NAC can be observed on the Al‐rich side (*x* from 0 to 0.045), which could be explained by the substitution of a small amount of Zn^2^⁺ in NAC. However, there is no linear behavior to confirm the existence of an extended solid solution, as all unit cell parameters remain constant when *x* increases further. Moreover, the Zn occupancy on the Al site was refined and is shown in Figure S5 (Supporting Information), demonstrating that the Zn occupancy remains near 0 for all compositions in the NAC phase. Substituting a Zn^2^⁺ ion (74 pm) into an Al^3^⁺ site (53.5 pm) requires significant distortion. Additionally, to maintain charge balance in the crystal, an extra Na⁺ ion would need to be intercalated into the structure. This is unfavorable for the stability of the NAC phase, as indicated by our previous DFT calculations of the substituted structure.^[^ ^51^ ^]^

On the Zn‐rich side, the diffractograms for nominal substitution degree *x* from 0.625 to 1 show no additional reflections aside from those of the NZC phase. Linear behavior of the lattice parameters *a* and *b* and unit cell volume is observed for *x* = 0.625 to 1, corroborating the formation of a solid solution.

From the refinement of the molar fraction of the two phases, we find a clear two‐phase behavior on the Al‐rich side (linear trend). From the extrapolation of the phase amounts, we find an intercept with the x‐axis at *x* = 0.66(3), indicating the limit of the miscibility gap. Thus, at low Zn content (0 ≤ *x* < 0.625), the system consists of a two‐phase mixture of the NAC and NZC‐type phases. When *x* exceeds the miscibility gap near 0.66(3), a pure solid solution in the NZC‐type phase appears. The Zn, Na1, and Na2 occupancies were refined for samples in this solid solution region (0.625 ≤ *x* ≤ 1) and are shown in Figure 4d,e. As discussed above, Na⁺ ions in the Na1 (Wyckoff *4a*) site are expected to be more mobile, and lower occupancies for Na1 compared to Na2 are observed across all compositions, suggesting that vacancies tend to form in the Na1 channel. For *x* < 0.75, the Na1 occupancy stabilizes close to 0.7, while the Na2 site is more (but not fully) occupied, close to 0.95. At *x* = 0.75, the difference between the two sites is smaller, with site occupancy of Na1 = 0.85(4) and Na2 = 0.91(4) (see Table S12, Supporting Information). Consistent with the solid solution limit near *x* = 0.625, Zn occupancies remain constant at 0.67(3) in the composition NZAC(*x* = 0.625), indicating that the crystallization of the NAC phase is energetically more favorable than the further substitution of Al^3^⁺ in the NZC‐type phase. Finally, Figure 4f displays the refined Zn content against the nominal total Zn content *x*. Since the refined Zn content considers all free parameters (molar amount of NAC‐ and NZC‐type phase and refined Zn content in each phase), the linear relationship with a slope of 1 along the diagonal strongly supports the reliability of our analysis.

Nonetheless, it is known that halide solid electrolytes synthesized by ball milling may contain an amorphous phase, which may play a crucial role in the conductivity.^[^ ^21^, ^32^, ^52^ ^]^ To verify this, the amorphous fraction of our samples has been determined using an internal reference. The analysis conducted with Al_2_O_3_ and Si internal references is reported in Figure S6 (Supporting Information). Indeed, our samples contain an amorphous phase ranging from 30% for NAC to 50% for NZC. The amount of amorphous phase is similar in all samples and increases from NAC to NZC.

**Figure 5:**
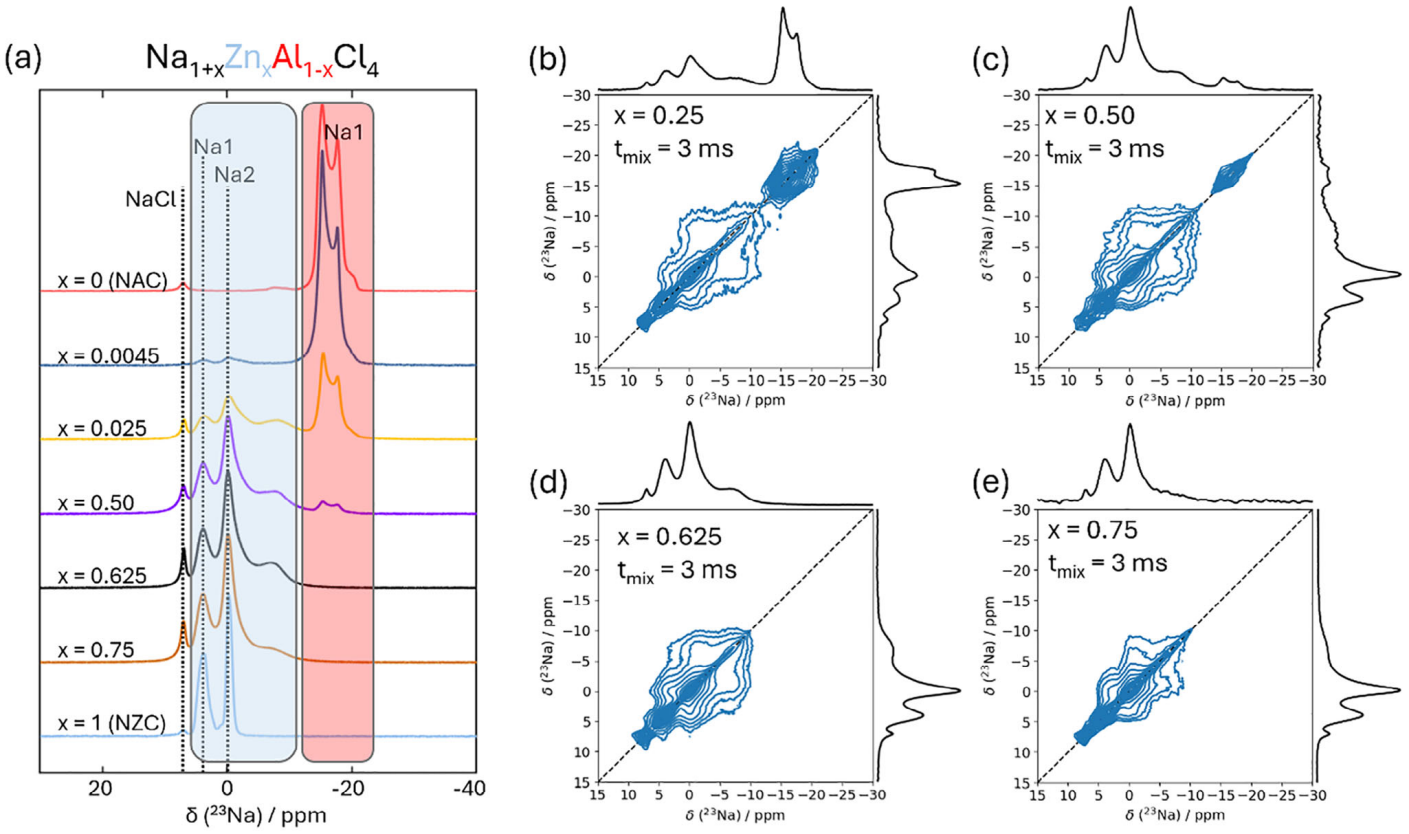
a) 1D ^23^Na MAS NMR spectra showing the central transitions for ball‐milled NZAC compositions with different substitution degrees (*x*). b‐e) 2D ^23^Na‐^23^Na EXSY NMR spectra collected for *x* = 0.25, 0.5, 0.625, and 0.75 compositions, respectively. For all exchange spectra, a mixing time of 3 ms was used.

The fact that our Rietveld analysis led to consistent Na and Zn contents in the crystalline part of the samples suggests that the crystalline and amorphous phases have similar compositions and possibly local structures. To verify that our structural analysis also holds at a more local scale, we employ ^23^Na MAS ss‐NMR, and the results are gathered in **Figure** **5**. The NMR data further confirm that NaAlCl_4_ does not appear to be doped with Zn^2+^: as shown in Figure 5a, the spectra of NZAC (*x* = 0, 0.045, 0.25, 0.5) contain the typical peak for Na1 in NaAlCl_4_ with unchanged peak position (−14.2 ppm) and quadrupolar line shape corroborating that the local environment of Na in NAC is the same for these compositions. With varying amounts of Zn (x), only the intensity of the peak changes following the phase ratios extracted from Rietveld refinements (Figure 5c). The ^23^Na MAS NMR spectrum of NZC (*x* = 1) displays two Na signals at 4.8 and 0.4 ppm with rather small apparent quadrupolar coupling constants of C_Q_ = 0.7 MHz (η∼0.5) and 0.5 MHz (η∼0), respectively, which are assigned to the Na1 and Na2 sites in NZC. These signals related to the NZC phase appear even at *x* = 0.045, indicating that Zn^2^⁺ prefers to form the NZC phase rather than substituting Al^3^⁺ in NAC. The characteristic peaks of NZC are distinguishable for *x* values ranging from 0.045 to 1. Moreover, for samples *x* = 0.75, *x* = 0.625, *x *= 0.5, and *x* = 0.25, a broad distribution of signals appears between 0 and −10 ppm, indicating the presence of disordered Na environments, which can be explained neither by pure NZC nor NAC. As we will discuss in the following, these environments are related to Na in the Al‐doped NZC phase.

2D ^23^Na‐^23^Na exchange (EXSY) MAS NMR spectra (Figure 5b–e) were conducted using a mixing time of 3 ms for NZAC compositions (*x* = 0.25, 0.5, 0.625, 0.75) to probe which Na sites exchange on the millisecond timescale. The spectra show no cross‐correlation (off‐diagonal) signals for the NAC phase (−14.2 ppm) in all compositions, but strong exchange (off‐diagonal intensity) is observed between the broad signal at −8 ppm and Na sites in the NZC phase. This indicates that on the ms‐timescale, Na^+^ exchange between disordered environments takes place in the NZC phase. Meanwhile, no Na^+^ exchange is observed between the NAC phase and the broad peak at −8 ppm, indicating that the broad peak is merely attributed to Na within the NZC‐type phase rather than as interface domain between NAC phase and NZC phase. The 2D ^23^Na‐^23^Na EXSY NMR spectrum of *x* = 0.75 compositions (Figure 5e) also shows some cross‐correlation intensity for the broad signals between 0 and −8 ppm. However, the intensity is significantly weaker compared to *x* = 0.25, 0.5, and 0.625 compositions. In summary, from the ^23^Na NMR data, we conclude that Zn^2+^ is substituted by Al^3+^ in the NZC phase, resulting in more disordered Na⁺ environments, potentially in Na1 and Na2 sites in the NZC structure. The 2D EXSY NMR spectra further indicate that the more mobile Na ions are mostly associated with the Na environments, which were introduced through the substitution of Zn^2+^ with Al^3+^.

Finally, DC polarization measurements (Figure S7a, Supporting Information) reveal that the electronic conductivity of all selected NZAC compositions is below 10⁻⁹ S cm^−1^. For the composition NAC and NZAC (*x* = 0.625), electrochemical stability windows ranging from 2.6 to 4.1 V versus Na^+^/Na and 2.6 to 3.9 V versus Na^+^/Na were measured, respectively, using the cell architecture Na_3_Sn|NPS|NZAC+carbon (Figure S7b,c, Supporting Information). Regarding the oxidation stability, the pure NAC sample shows a higher onset at 4.1 V versus Na^+^/Na compared to NZAC (*x* = 0.625); the onset value of NAC is in complete agreement with that reported in previous literature,^[^ ^27^ ^]^ while two oxidation peaks are observable during the first oxidation sweep of NZAC (*x* = 0.625), which clearly points to Zn being responsible for the decreased oxidation stability. The reduction stability window of NAC and NZAC is not as low as the theoretical value (1.494 V as computed by first‐principles calculation in the literature),^[^ ^27^ ^]^ which suggests the need for a reduction‐resistant interlayer between the anode and NZAC samples in cells.

### 2.4 Ionic Conductivity and Na Transport Mechanism

**Figure 6:**
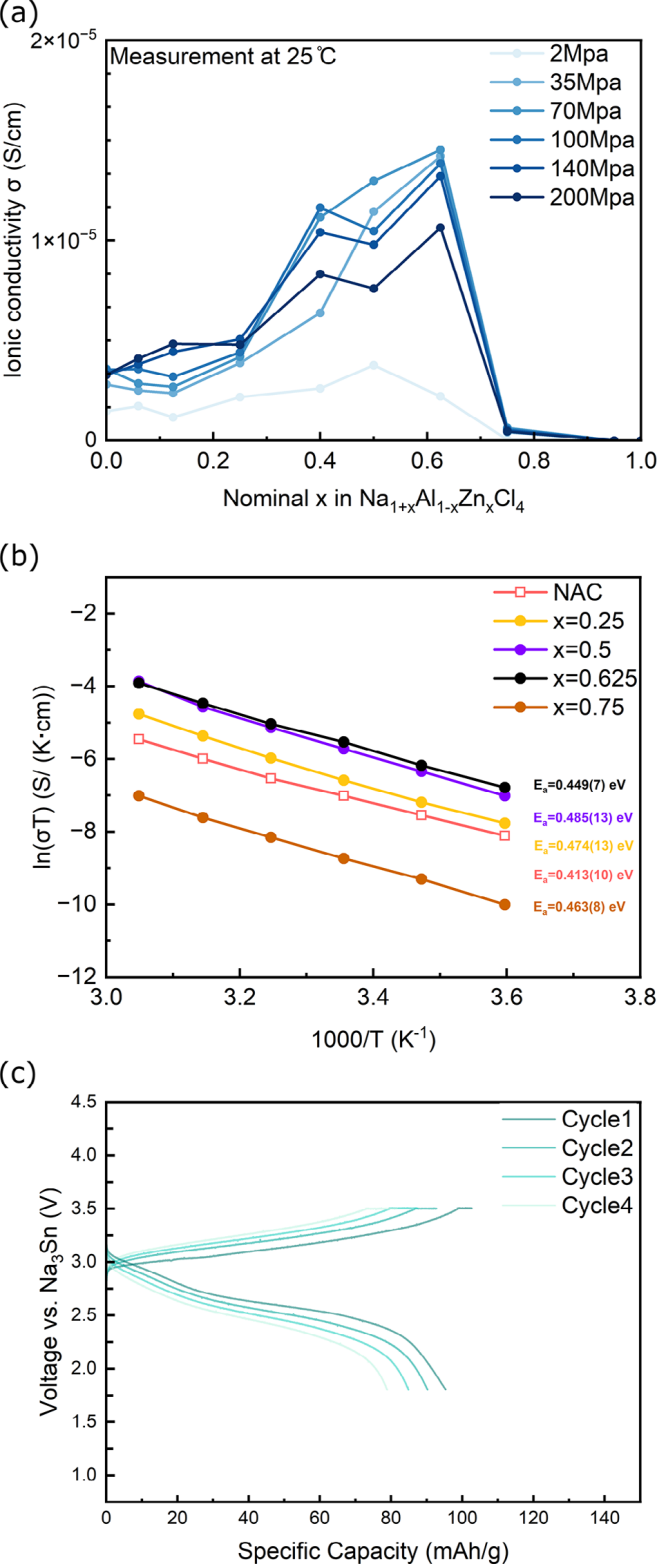
a) Ionic conductivities of Na_1+_ *_x_* Zn*_x_* Al_1‐_ *_x_* Cl_4_ at 25 °C as a function of applied pressure. b) Arrhenius plots of Na_1+_ *_x_* Zn*_x_* Al_1‐_ *_x_* Cl_4_ (*x* = 0, 0.25, 0.5, 0.625, and 0.75) at different temperatures, measured under pressure of 140 MPa. NAC phases are denoted as hollow squares due to the different Na^+^ pathways compared to other NZC‐type phases. c) Charge‐discharge profiles and cycling performance of all‐solid‐state battery composed of NZAC (*x* = 0.5) catholyte at 0.1 C, 25 °C, 70 MPa. 0.1C = 0.0956 mA cm^−2^.

**Figure** **6**a shows the ionic conductivities obtained from EIS measurements for all Na₁₊_x_Zn_x_Al₁₋_x_Cl₄ nominal compositions under different applied pressures. The same data are also shown on a logarithmic scale in Figure S8 (Supporting Information). For *x* = 0, the pure NAC composition exhibits an ionic conductivity of 1.5 × 10⁻⁶ S cm^−1^ at 2 MPa. Increasing the pressure to 200 MPa makes little difference, i.e., the conductivity only reaches 3.3 × 10⁻⁶ S cm^−1^ at 200 MPa. As *x* increases, the ionic conductivity shows an upward trend, reaching a maximum value of 1.5 × 10⁻⁵ S cm^−1^ at 35 MPa for Zn content in the range of 0.5 ≤ *x* ≤ 0.625. It should be noted that 2 MPa is not enough to observe a significant increase in conductivity, whereas 35 MPa enables this improvement. Higher pressures up to 200 MPa offer no additional benefit; on the contrary, a slight decrease in conductivity is observed.

To understand the origin of the increased conductivity, we refer to our findings on the material's composition. We concluded that NaAlCl_4_ is undoped with Zn^2^⁺. Interestingly, the highest conductivities are achieved for the composition Na_1.625_Zn_0.625_Al_0.375_Cl₄, close to the edge of the miscibility gap. Since NaAlCl_4_ does not show any improvement in conductivity even at 35 MPa, one may hypothesize that the increase in conductivity arises from either the introduction of sufficient vacancies in the Na1 sites of the NZC‐type structure, allowing for effective Na hopping, or the influence of the amorphous phase in the samples. Previous studies have demonstrated that the amorphous phase plays a dominant role in ionic conduction in some sodium metal halide SSEs. These works discussed three ways through which amorphization may lead to improved ionic conductivity: i) introducing O^2−^ to substitute Cl^−^ in NaMCl_6_ (M = Ta, Nb),^[^ ^36^, ^37^, ^39^, ^41^ ^]^ Na_2_MCl_6_ (M = Hf, Zr)^[^ ^38^, ^53^ ^]^ or NaAlCl_4_^[^ ^35^, ^42^ ^]^ ii) Na‐deficiency in Na_2_₋_x_MX_6_₋_x_ (X = F or Cl)^[^ ^32^, ^54^ ^]^ iii) long ball‐milling to destabilize the crystalline structure.^[^ ^52^ ^]^ Notably, our samples do not conform to any of the three conditions outlined above, as they do not contain oxygen or halide vacancies, and all samples contain a similar amorphous content (≈30%–50%, Figure S6, Supporting Information). The amorphous content gradually increases with *x* up to *x *= 0.5 and *x* = 0.625, where the NZAC samples exhibit optimal ionic conduction. When *x* further increases to *x* = 0.75, the amorphous fraction remains comparable, but the ionic conductivity declines by an order of magnitude. In the pure NZC phase, which also exhibits a slightly higher amorphous content, the ionic conductivity essentially disappears. Therefore, we can conclude that the 2‐phase system Na₁₊_x_Zn_x_Al₁₋_x_Cl₄ shows no intuitive correlation between ionic conductivity and amorphous content.

Additionally, to further investigate the effect of a potential amorphous phase contribution in NZAC samples, post‐annealing on NZAC (*x* = 0.625) was also conducted. The material was subjected to an 8‐hours postannealing treatment at 90 °C under 2 MPa. The evolution of the amorphous fraction, ionic conductivity, and ^23^Na NMR spectra are depicted in Figure S9 (Supporting Information), and the refinement details are listed in Table S11b (Supporting Information). An increased crystallite size (36 to 50 nm after annealing) was derived from XRD Rietveld refinement, while the amorphous fraction of NZAC (*x* = 0.625) remains constant at approximately 50% after annealing, indicating the thermal stability of the ball‐milled compound. Importantly, the ionic conductivity decreased by a factor of three (Figure S9a, Supporting Information). As the amorphous fraction did not change, one can conclude that it is not correlated to the ionic conductivity, while on the other hand, the larger crystallites can be concluded to have a negative impact on the measured conductivity. Furthermore, we compared 1D ^23^Na MAS NMR spectra before and after post‐annealing at 90 °C for 8 h (Figure S9b, Supporting Information). The results reveal that multiple signals become sharper, especially visible for the peak at 4 ppm and the broad peaks at low chemical shifts between −6 and −10 ppm. The latter broad peak reflects two distinguishable peaks, indicating increased ordering of Na in the NZC‐type phase after annealing. Furthermore, 2D ^23^Na‐^23^Na EXSY NMR spectra (Figure S9b,c, Supporting Information) show weaker Na ion exchange after annealing, which aligns with the results of EIS measurement in Figure S9a (Supporting Information). Thus, we conclude that the solid solutions are stable upon annealing and that the reduced ionic conductivity is associated with the NZC‐type phase featuring larger crystallites with more ordered Na environments.

To gain understanding, EIS measurements were conducted at a fixed pressure (140 MPa) while varying the temperature. Arrhenius plots for all Na_1+_ *_x_* Zn*_x_* Al_1‐_ *_x_* Cl_4_ compositions are presented in Figure 6b. As expected, ln(σT) versus 1/T exhibits a linear behavior within the probed temperature range. The activation energy E_a_ was calculated from the following Arrhenius relation:^[^ ^29^ ^]^

(1) $$\sigma T=\sigma_{0}exp\left(-\frac{E_{a}}{k_{B}T}\right)$$

E_a_ represents the activation energy, σ_0_ the Arrhenius pre‐factor, and k_B_ the Boltzmann constant. All Na_1+_ *_x_* Zn*_x_* Al_1‐_ *_x_* Cl_4_ (*x* ≥ 0.25) compositions exhibit activation energies in the range of 0.449–0.485 eV, which is higher than pure NAC (0.413 ± 0.010 eV). This apparent contradiction indicates that the NAC and NZC‐type phases involve different Na^+^ hopping mechanisms as well as distinct energy barriers. As a result, despite exhibiting a lower σ compared to the optimal NZAC (*x* = 0.625) samples, the NAC phase has a lower E_a_. For the samples that possess a predominant NZC‐type structure, such as *x* = 0.625 and *x* = 0.75, only a slight difference in E_a_ is present, which indicates the same hopping mechanism. The sample *x* = 0.625, which displays the highest conductivity, also shows the lowest activation energy of 0.449 ± 0.007 eV among NZC‐type structures. However, the significant difference in σ between the NAC and NZC‐type phases cannot be explained by the activation energies; hence could be attributed to the prefactor σ_0,_ which is an empirical parameter related to charge carrier concentration. In fact, for the same type of structure, the ionic conductivity (σ) can also be defined as:

(2) $$\sigma =n_{Na^{+}}\cdot q_{Na^{+}}\cdot \mu_{Na^{+}}$$

here, n represents the number of charge carriers (Na^+^) per unit volume, q is the charge of Na^+^, and μ is the mobility of Na^+^. When the *x* value decreases from *x* = 0.75 to *x* = 0.625, more Na^+^, particularly in the Na1 site, will be replaced by vacancies, possibly activating the participation of adjacent Na^+^ ions in the transport mechanism, thereby increasing the density of charge carriers $n_{Na^{+}}$. Moreover, due to the two‐phase behavior observed when *x* is between 0 and 0.5, no typical trade‐off relationship between n and μ,  as often seen in ionic conductors, can be established in this case.^[^ ^32^, ^33^ ^]^

As proof of feasibility, the NZAC (*x *= 0.5) was integrated as the catholyte into all solid‐state batteries (ASSBs). The cell with a configuration of Na_3_Sn|Na_3_PS_4_|NaCrO_2_ cathode composite was assembled with Na_3_PS_4_ as an interlayer to prevent direct contact between Na_3_Sn and NZAC SSE. The ASSB applying NZAC as catholytes was cycled at 25 °C, the charge‐discharge profiles of the first four cycles are shown in Figure 6c. At 0.1C current, the NZAC catholyte delivers a first cycle discharge capacity of 93 mAh g^−1^ and an initial Coulombic efficiency (ICE) value of 92.9%, followed by four cycles with CE > 96%. These results are encouraging, yet the cell polarization is still high, and future work will be devoted to improving the cell design and the related performance.

In summary, introducing sufficient vacancies within the NZC‐type phase, while maintaining a high phase fraction, is key to achieving the highest ionic conductivity in NZAC samples. Therefore, optimal compositions are close to the solid‐solution boundary near *x *= 0.625. In the following, we further support our conclusions and clarify the conductivity mechanism by MD and NEB simulations, coupled with simulations of ss‐NMR spectra.

### 2.5 Ionic Conductivity by Molecular Dynamics and ss‐NMR

In the previous sections, we showed how samples with *x* = 0.5 and 0.625, based on the Na_2_ZnCl_4_ structure, exhibit significantly enhanced conductivity with respect to the end member NZC. We also showed that this does not correlate with the amorphous phase present in the samples; rather, we observed a novel signal in the ss‐NMR spectra of these intermediate samples (Figure 5a), whose origin and correlation with conductivity is yet to be clarified. The origin of the significantly enhanced ionic conductivity is explored by theoretical MD and NEB simulations on machine‐learned potential MACE‐MP‐0/D3(BJ)‐level and with theoretical NMR calculations on PBE/D3(BJ)‐level and experimental ss‐NMR measurements. Herein, in the NZC structure, Na atoms in the Na1 sublattice surrounded by Al^3+^ tetrahedra are denoted as Na1(Al), while Na atoms in the Na1 sublattice surrounded by both Al^3+^Cl_4_ tetrahedra and Zn^2+^Cl_4_ tetrahedra are denoted as Na1(Zn/Al); the same nomenclature also applies to Na atoms in the Na2 sublattice.

**Figure 7:**
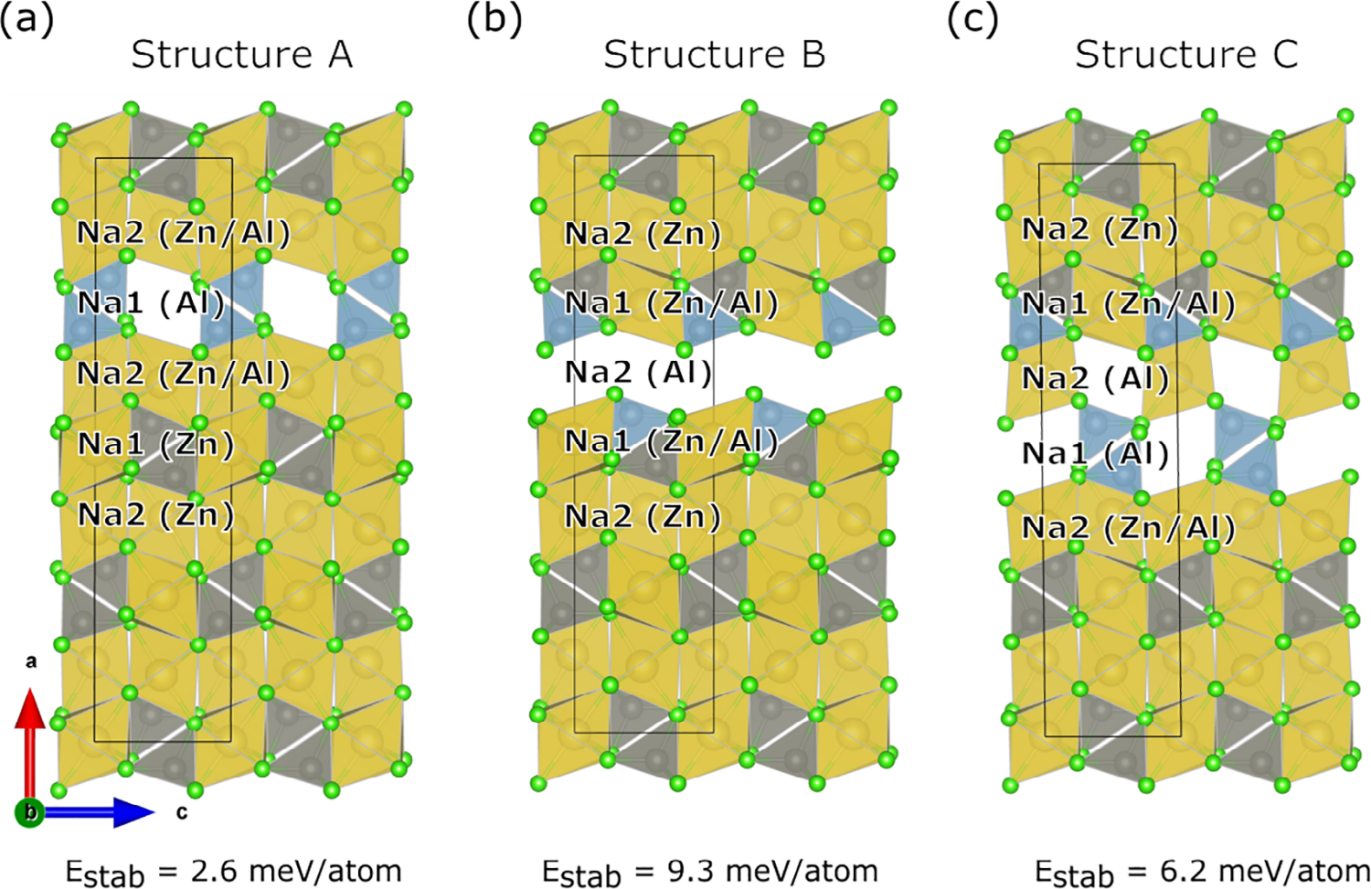
a) Geometry of Na_1.75_Zn_0.75_Al_0.25_Cl_4_ with vacant Na1(Al) layer, b) Geometry of Na_1.75_Zn_0.75_Al_0.25_Cl_4_ with vacant Na2(Al) vacancies, and c) Geometry of Na_1.625_Zn_0.625_Al_0.375_Cl_4_, all viewed along [010]. Energies calculated with MACE‐MP‐0/D3(BJ) were given, representing the stability of such structures versus the ternary chlorides Na_2_ZnCl_4_ and NaAlCl_4_ (energy above the hull).

The mobility of the Na^+^ in Na_1.75_Zn_0.75_Al_0.25_Cl_4_ and Na_1.625_Zn_0.625_Al_0.375_Cl_4,_ based on its NZC‐type structure, was first evaluated with MD simulations. Two distinct model configurations of Na_1.75_Zn_0.75_Al_0.25_Cl_4_, either containing only Na1(Al) vacancies (**Figure** **7**a, denoted as structure A) or only Na2(Al) vacancies (Figure 7b, denoted as structure B), were chosen as representatives for the layer‐like structure type that was calculated previously to be most stable.^[^ ^51^ ^]^ The model shown in Figure 7a was taken as a base for the model of structure C with composition Na_1.625_Zn_0.625_Al_0.375_Cl_4_ (Figure 7c), whose Na site occupations are close to the ones obtained from X‐ray diffraction results (Na1 Occ. = 0.74(4), Na2 Occ. = 0.94(4), Figure 4d). Here, structure C is composed of half‐filled Na2(Zn/Al) adjacent to the entirely vacant Na1(Al) layer. It is worth emphasizing that all the model structures have somewhat idealized site occupancies, close but not identical to the experimentally observed ones, which are applied for the sake of simulation simplicity. Furthermore, simulation temperatures between 475 K and 700 K were chosen to accelerate the Na diffusion process to timescales that are observable on a picosecond time scale. Consequently, deviations from the experimental measurements at room temperature are possible.

**Figure 8:**
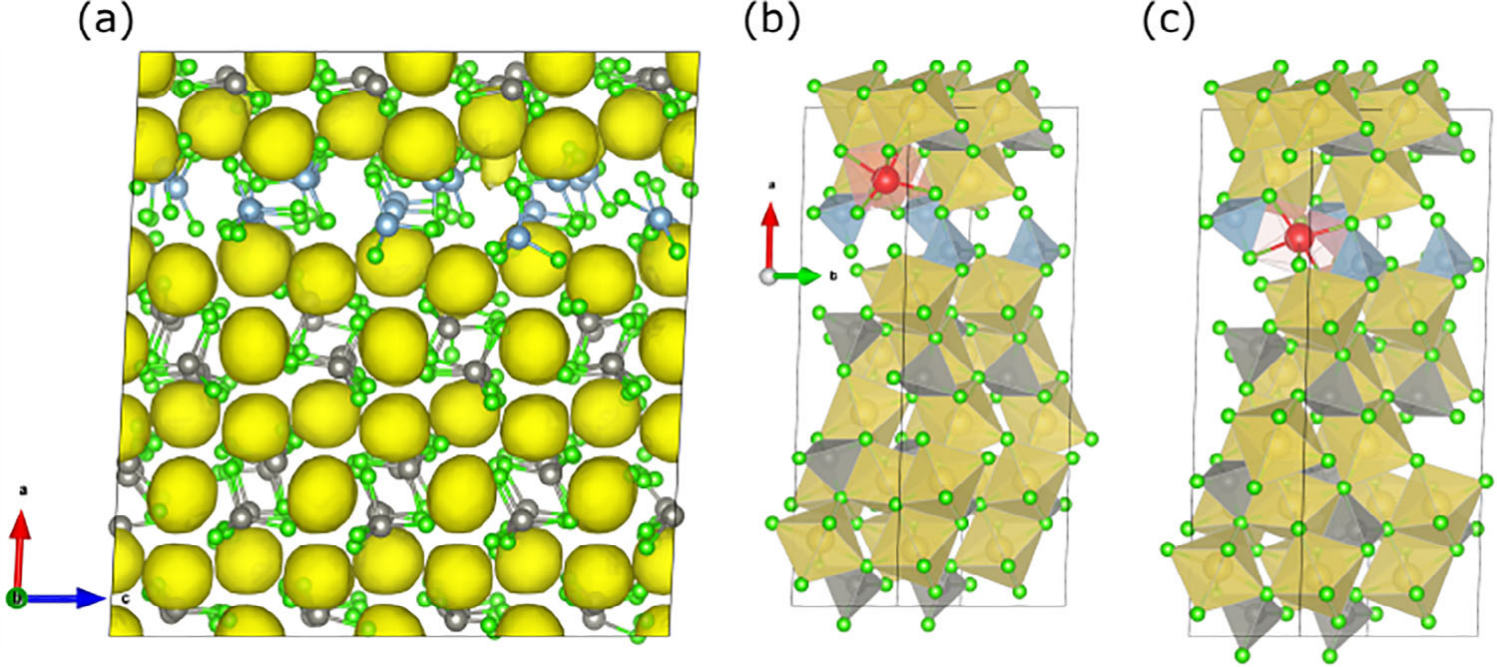
a) Simulated Na density plot for the NPT‐MD simulation of Na_1.75_Zn_0.75_Al_0.25_Cl_4_ (structure A) at 700 K with an isosurface cutoff of 5%. b) Starting structure and c) end structure for the NEB of a Na^+^ migration from an occupied Na2‐site to an unoccupied Na1‐site in the Na1(Al) layer.

For the most stable Na_1.75_Zn_0.75_Al_0.25_Cl_4_ structure with solely Na1 vacancies (Figure 7a), no ion movement between sites was observed by MD simulations. The distribution of sodium in structure A at 700 K is visualized in **Figure** **8**a, and the mean square displacement (MSD) at 500, 600, and 700 K is shown in Figure S10a (Supporting Information). The only movement that was found takes place along the *a*‐axis locally restricted between vacant Na1 positions in the Na1(Al) layer and the adjacent Na2 positions. On the time scale of the simulation, no long‐range movement occurs along the *a*, *b*, or *c* axes, which would be needed for ionic conductivity. As a side remark, for pure Na_2_ZnCl_4,_ not a single sodium ion jump was observed within the MD simulation time of 75 picoseconds at T < 800 K.

To better understand the lack of mobility of Na^+^ ions in structure A, NEB calculations were carried out for the migration of Na^+^ from a Na2 site into a neighboring empty Na1(Al) layer (Figure 8b,c). A 1×2×2 supercells of the structure in Figure 8a were employed to reduce the artificial strain on the images of the NEB calculation. The NEB yields an activation energy of 0.62 eV for the movement of Na^+^ from the Na2 layer into the Al layer, indicating that this migration path is kinetically inhibited.

**Figure 9:**
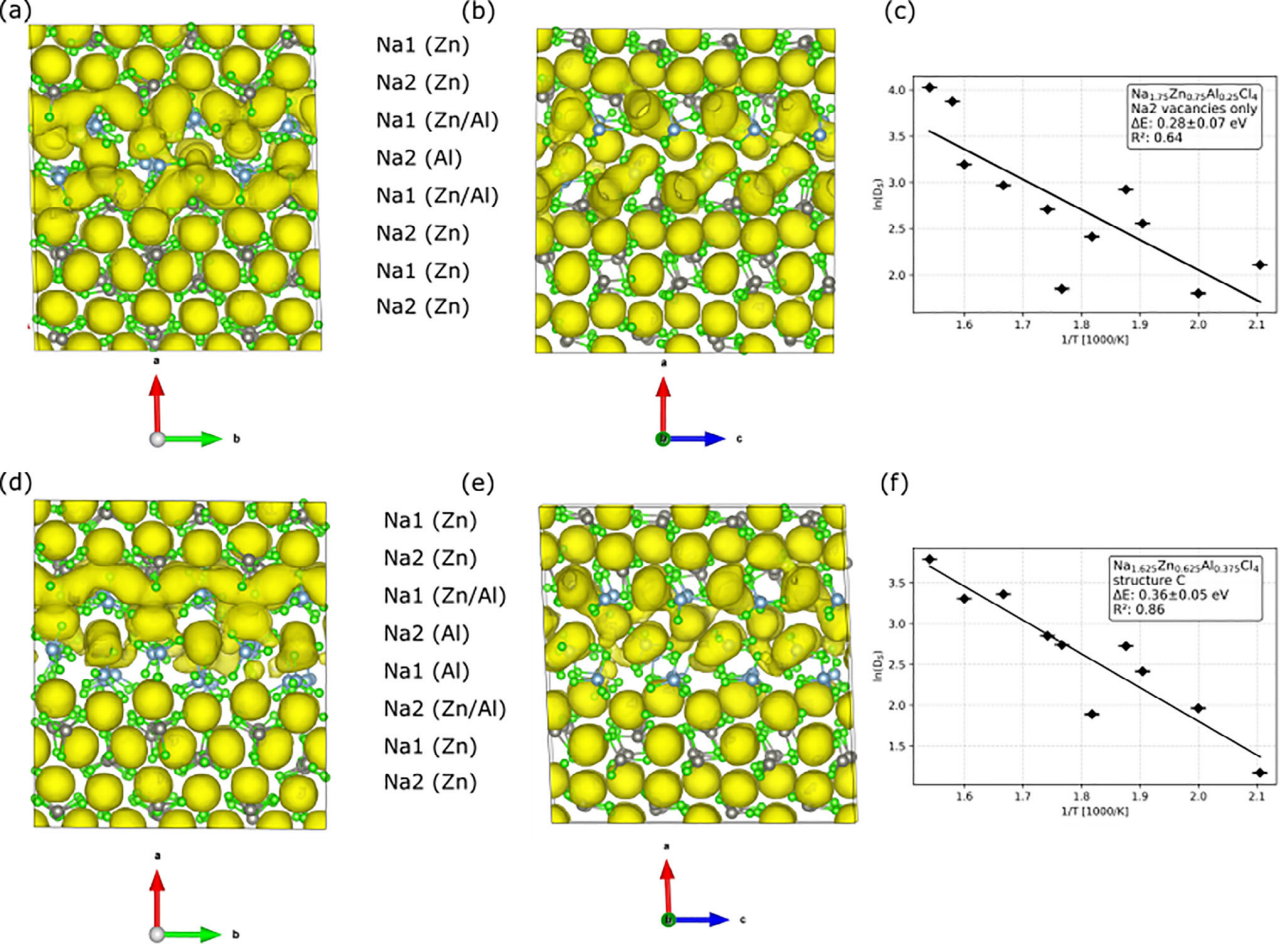
Simulated Na density plot for the NPT‐MD simulation of Na_1.75_Zn_0.75_Al_0.25_Cl_4_ (structure B) and Na_1.625_Zn_0.625_Al_0.375_Cl_4_ (structure C) at 650 K with an isosurface cutoff of 5%. a) Structure B viewed along [001] with Na1 and Na2 positions labeled according to whether they are in the vicinity of ZnCl_4_^2−^ or AlCl_4_^−^ or both. b) Structure B viewed along [010]. c) Arrhenius plot of the self‐diffusion constants obtained from the NPT MD simulation of structure B between 475 and 650 K. d) Structure C viewed along [001]. e) Structure C viewed along [010] with Na1 and Na2 positions labeled according to whether they are in the vicinity of ZnCl_4_^2−^ or AlCl_4_^−^ or both. f) Arrhenius plot of the self‐diffusion constants obtained from the NPT MD simulation of structure C between 475 and 650 K.

Structure B has only Na2 vacancies. This is only a hypothetical structure, as it is energetically less stable than configuration A (Figure 7), and we experimentally observe the Na1 site to be less occupied than Na2. Interestingly, however, Na^+^ migration was observed in the MD simulation at temperatures as low as 475 K (as shown in the simulated density plots of **Figure** **9**a,b, MSD plot in Figure S10b in Supporting Information), while no diffusion of Cl^−^ was observed (Figure S10c in Supporting Information). The main diffusion occurs between edge‐sharing Na1 sites in the mixed Al/Zn layers along the *b* axis. This migration is akin to Li^+^ migration in isostructural LiFePO_4_.^[^ ^48^, ^49^ ^]^ It is facilitated by the appearance of vacancies in the Na1(Zn/Al) layer through the spontaneous diffusion of Na1(Zn/Al) ions into the adjacent vacant Na2(Al) layer, in this case, Na2 vacancies function as intermediate sites that are crucial for Na1(Al/Zn) hopping to another Na1(Al/Zn) site.

Figure 9c shows an Arrhenius plot for the calculated self‐diffusion constants of structure B, while MSDs are depicted in Figure S10b (Supporting Information). The activation energy for the self‐diffusion of the Na ions in structure B is calculated to be 0.28 ± 0.07 eV. This is below the results from experimental ionic conductivity measurements in Figure 6b. The deviation likely originates, as mentioned before, from the simulated system only representing a simplified, idealized model at elevated temperatures rather than a complex, realistic composition. Finally, according to the XRD refinements in Figure 4d, the vacancies are neither exclusively located at the Na1 site nor the Na2 site, which implies the co‐existence of structures A and B to some extent. This leads to a much higher ionic conductivity of Na_1.75_Zn_0.75_Al_0.25_Cl_4_ composition than pure Na_2_ZnCl_4,_ which is observable in Figure S8 (Supporting Information).

In the next step, we investigated how increasing the number of vacancies affects Na^+^ mobility based on structure C (Figure 7c) with composition Na_1.625_Zn_0.625_Al_0.375_Cl_4_. Similar to structure B, a significant Na^+^ ion mobility was observed in the Na1(Zn/Al) layer according to MD simulations at 650 K (MSDs in Figure S10e in Supporting Information). In contrast, Cl^−^ ions do not diffuse at any temperature (Figure S10d in Supporting Information). For structure C, the Na^+^ density plot in Figure 9d,e shows similar patterns as the plot for structure B in Figure 9a,b, featuring a channel along the *b* axis and a zig‐zag path along the *c* axis across corner‐sharing Na octahedra, which is not observed for LiFePO_4_. Na ions move predominantly along *b*, as expected, even though diffusion along the other directions is also observed, as shown in the 1D‐MSD plot in Figure S11 (Supporting Information). An activation barrier of 0.36 ± 0.05 eV is obtained for structure C from the Arrhenius plot (Figure 9f) of the Na self‐diffusion constants (MSDs in Figure S10e, Supporting Information). The activation energy and diffusion rates were found to be comparable to the ones calculated for structure B but lower than the experimental findings of E_a_ = 0.449 ± 0.007 for *x* = 0.625. Further details on the NEB simulation and stability comparisons between MACE‐MP‐0/D3(BJ) and PBE/D3 (BJ) can be found in the Supporting Information in Figures S12 and S13 and Tables S14 and S15 (Supporting Information).

The main qualitative difference between structures B and C is that C possesses a more significant conduction channel in the *b* direction (Figure 9a,d, isosurface cut‐off is always 5%). This difference originates from the higher Na^+^ vacancy concentration in structure C, which provides more efficient hopping, i.e., more Na^+^ charge carriers are activated across both Na1 and Na2 sites during conduction. It should also be noted that structure C in Figure 7c, with full Na^+^ occupation in the Na1(Zn/Al) layer only, represents the starting configuration. However, local diffusion along the *a* direction is also observed during the simulation (Figure 9d,e), which introduces vacancies into the Na1(Zn/Al) layer. This, in turn, increases the Na1 occupancy in the Na1(Al) layer, which can only occur via exchange with Na2 sites in the Na2(Al) layer.

In brief conclusion, MD simulations and NEB calculations of the model systems for Na_1.75_Zn_0.75_Al_0.25_Cl_4_ and Na_1.625_Zn_0.625_Al_0.375_Cl_4_ reveal that the enhanced conductivity of Na_1+_ *_x_* Zn*_x_* Al_1‐_ *_x_* Cl_4_ originates from the introduction of both Na1 and Na2 vacancies, which can exchange via Na1 sites in mixed Al/Zn environments. In this way, the active charge carrier concentration, i.e., mobile Na ions, is increased compared to the Na_2_ZnCl_4_ structure. Especially, the interplay between Al‐dominated regions and Zn‐dominated regions plays a critical role: as visible in structure C (Figure 9d,e), Na^+^ in the Na1(Al) layer (i.e., in pure Al environments) does not diffuse via the same channel in the *b* direction as in the Na1(Zn/Al) layer. This is likely due to the modified local geometry, since the AlCl_4_^−^ tetrahedra are significantly smaller than the ZnCl_4_^2−^ tetrahedra. On the other hand, the Na hopping between Na1(Zn/Al) and Na2(Al) is also critical since Na2(Al) can be partially filled while allowing the introduction of vacancies into Na1 (Zn/Al) sites. The latter is responsible for enhanced ionic conduction of Na_1.625_Zn_0.625_Al_0.375_Cl_4_.

**Figure 10:**
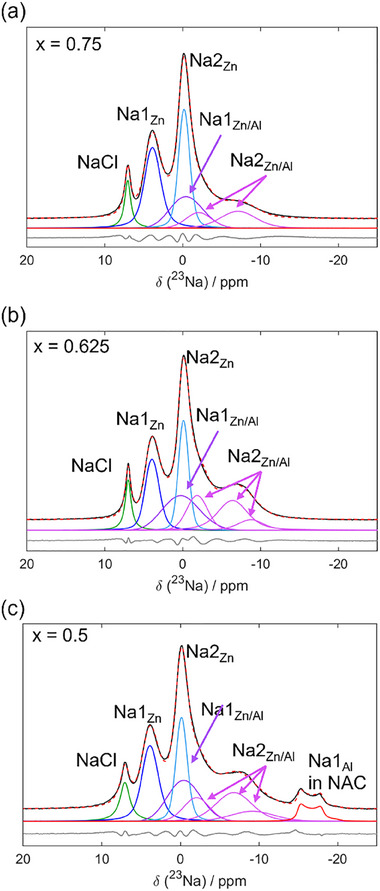
Deconvolution of the central transition of the 1D ^23^Na MAS NMR spectra for ball‐milled NZAC composition with different substitution degrees a) *x *= 0.75, b) *x = *0.625, and c) *x* = 0.5. The experimental spectrum is shown in black, the sum of the deconvolution of the individual signals as a dashed red line, and the difference in grey below.

Comparing our simulations to the experimental solid‐state NMR spectra for the samples with compositions of *x *= 0.5, *x* = 0.625, and *x* = 0.75 may corroborate the mechanisms at play. Therefore, first, the number and nature of ^23^Na sites present in these three samples were extracted from 2D ^23^Na Multiple‐Quantum Magic Angle Spinning (MQMAS) NMR experiments (Figure S14; Table S16, Supporting Information), where the signals are separated according to their isotropic chemical shift in the indirect dimension (y axis), while the direct dimension contains both, the chemical shift and the quadrupolar interaction. The MQMAS spectra show the signals for Na in typical Na1 (5 ppm) and Na2 (0.4 ppm) environments from NZC with small quadrupolar couplings (< 0.7 MHz). However, the spectra reveal an additional Na signal at around 1–3 ppm with a significant quadrupolar broadening with C_Q_∼1 MHz and additional disorder. Furthermore, the MQMAS spectra demonstrate a clear difference between the *x* = 0.5 and *x* = 0.625 compared to the *x* = 0.75 composition: while for *x* = 0.5 and *x* = 0.625 a distribution of Na signals with small C_Q_ (< 0.5 MHz) is observed within an isotropic shift range as wide as 10 ppm (δ_iso _= 0 and ≈−10 ppm), the chemical shift range for *x* = 0.75 is restricted between 0 and −6 ppm. This indicates that different Na environments form with more Al substitution in the direct environment. We used the extracted Na sites from the MQMAS spectra to fit the 1D ^23^Na MAS NMR spectra of all three compositions (**Figure** **10**, Tables S17 and S18, Supporting Information).

To assign the different experimentally observed ^23^Na signals to different Na sites and local environments, we calculated the expected NMR parameters for the DFT structures of NaAlCl_4_, Na_2_ZnCl_4_, as well as Na_1.75_Zn_0.75_Al_0.25_Cl_4_ with solely Na1 or solely Na2 vacancies, the two energetically favored structures.^[^ ^51^ ^]^ The results (summarized in Figure S15, Table S19, Supporting Information) are in good agreement with the experimental signals for the *x* = 0.75 composition, considering the degree of disorder and strain from the ball milling syntheses, and therefore allow for an assignment of the ^23^Na signals. Na1 and Na2 sites in pure Zn environments exhibit chemical shifts that are very close to the ones calculated and observed for NZC. However, as soon as mixed Al/Zn environments occur, the values are shifted to lower chemical shifts. Hence the experimentally observed, broad signal at ≈2 ppm with a C_Q_ of ≈1 MHz is assigned to Na1 sites with mixed Al/Zn environment (Na1(Zn/Al)), while Na2 sites with mixed Al/Zn environments are shifted to values below 0 and −5.5 ppm (Na2(Zn/Al)). Quadrupolar couplings of the latter are higher from DFT calculations than experimentally observed, which could either be caused by the disorder in the system or by partial motional averaging, which may take place by local Na^+^ motion as indicated from the MD calculations and 2D ^23^Na EXSY NMR spectra (Figure 5b–e). Using the deconvolution of the 1D ^23^Na MAS NMR spectrum (Figure 10a), we can estimate a NZAC composition of Na1_0.88_Na2_0.87_Zn_0.75_Al_0.25_Cl_4_ for the nominal *x* = 0.75 sample, which is in good agreement with the Na1/Na2 occupancies derived from XRD analyses (Figure 4d). Thus, we conclude that in the case of *x* = 0.75, both layers of Na1 or Na2 vacancies form during the high‐energy ball milling synthesis. For structures with solely Na1 vacancies (structure A), Na exchange between full and empty layers is kinetically inhibited by a high activation barrier, as shown above by the NEB calculations (Figure 8a–c). However, for solely Na2 vacancies (structure B), Na jumps between Na1(Zn/Al) and Na2(Al) layers are possible. Thus, the experimentally observed limited Na^+^ ionic conductivity (Figure S8, Supporting Information) is explained by the coexistence of both layers of Na1 or Na2 vacancies and by the insufficient total vacancy density. This is also reflected in the limited cross‐signal intensity of the corresponding 2D EXSY NMR spectrum in Figure 5e. However, turning to the *x* = 0.5 and *x* = 0.625 samples, we do observe a distribution of an additional ^23^Na signals with small chemical shifts between −5 and −10 ppm, which were also not predicted by DFT calculations of the model compositions. Thus, we assign the additional signal intensity to Na in environments in NZC‐type structures that are, however, similar to those in NaAlCl_4_, i.e., Al‐rich and closer to seven‐fold coordinated by Cl^−^. From the fits of the 1D ^23^Na MAS NMR spectra (Figure 10b,c) of the nominal *x* = 0.625 and *x* = 0.5 compositions, we derive NZAC phases with compositions of Na1_0.70_Na2_0.97_Zn_0.67_Al_0.33_Cl_4_ and Na1_0.75_Na2_0.94_Zn_0.69_Al_0.31_Cl_4_, respectively. Both are again in good agreement with the Na1/Na2 occupancies derived from XRD analyses (Figure 4d).

The MD simulations indicate that Na1 sites in mixed Al/Zn and pure Al environments, as well as Na2 sites in Al‐rich environments, are crucial for ionic transport. This is further supported by the ^23^Na‐^23^Na EXSY MAS NMR spectra (Figure 5d), where the strongest exchange with significant intensity is observed between Na2 and Na1 sites, both in mixed Al/Zn environments. This is completely consistent with the results from MD simulations of structure C (Figure 9d,e). To summarize, the aliovalent substitution of Al^3+^ for Zn^2+^ (*x* > 0.25) leads to the formation of preferred Na1 vacancies (also resulting in Na2 sites in Al‐rich environments) but requires the formation of additional Na2 vacancies for charge compensation. These additional Na2 vacancies significantly facilitate the Na^+^ movement between Na1 and Na2 layers, which is in accordance with the diffusion pathways found in the MD simulations and experimental EXSY NMR measurements.

## 3 Conclusion

In this work, a series of Na_1+_ *_x_* Zn*_x_* Al_1‐_ *_x_* Cl_4_ samples were synthesized using a mechanochemical method. The crystal structures were primarily studied through XRD and related Rietveld refinement. The local Na environment in pure NaAlCl_4_ (space group *P*2_1_2_1_2_1_) was deciphered with the help of sXRD and ss‐NMR spectroscopy, and only one Na site was identified. The solubility of Zn^2^⁺ and Al^3^⁺ in the two end members was investigated, with support from 2D EXSY‐NMR. No evidence of Zn solubility into NaAlCl_4_ is observed. A solid solution forms instead in the NZC phase with Al^3^⁺ and Zn^2^⁺ occupying the same tetrahedral site (Wyckoff position *4c*). However, this solid solution has a limit: when the Zn content is less than *x *= 0.66(3), the NaAlCl_4_ phase without Zn^2^⁺ doping appears, ultimately indicating a miscibility gap. The ionic conductivities of Na_1+_ *_x_* Zn*_x_* Al_1‐_ *_x_* Cl_4_ were measured via EIS under varying temperatures and pressures. The highest values of ionic conductivity (35 MPa, 25 °C) reach 1.5 × 10⁻⁵ S cm^−1^, and were observed for Zn contents in the range of 0.5 ≤ *x* ≤ 0.625. This increase in conductivity was investigated by computational methods and ss‐NMR spectroscopy, and it was attributed to the rise in vacancy concentration and cation disorder in the Al^3^⁺‐doped Na_2_ZnCl_4_ phase. The presence of vacancies in both Na1 and Na2 sites was shown to be crucial. This behavior was further modelled using MD simulations, which showed good agreement with the experimental NMR results. In conclusion, we demonstrate that samples close to the phase boundary at *x* = 0.625 appear to be ideal for achieving the highest conductivity in this material family. Here, the vacancy concentration is high enough, and the sample is monophasic. At higher *x*, the vacancy concentration is too low, while at lower ones the amount of secondary NaAlCl_4_ phase increases and lowers the overall conductivity. Ultimately, we also demonstrate a proof‐of‐concept solid‐state sodium cell using a NZAC sample as catholyte. This work introduces a novel class of SSE based on the NZC structure and provides insights into the correlation between composition, crystalline structure, and ionic conduction pathways. Finally, it highlights the contributions of NMR and MD simulation to studying metal halide SEs and the Na transport mechanisms within.

## Conflict of Interest

The authors declare no competing financial interest.

## Supporting information



Supporting Information

## Data Availability

The data that support the findings of this study are available from the corresponding author upon reasonable request.

## References

[1] J.‐M. Tarascon , M. Armand , Nature 2001, 414, 359. 11713543 10.1038/35104644

[2] J. B. Goodenough , K.‐S. Park , J. Am. Chem. Soc. 2013, 135, 1167. 23294028 10.1021/ja3091438

[3] M. A. Hannan , M. M. Hoque , A. Mohamed , A. Ayob , Renew. Sustain. Energy Rev. 2017, 69, 771.

[4] Lithium – Price – Chart – Historical Data – News. https://tradingeconomics.com/commodity/lithium (accessed: October 2024).

[5] J. Janek , W. G. Zeier , Nat. Energy 2016, 1, 16141.

[6] H. S. Hirsh , Y. Li , D. H. S. Tan , M. Zhang , E. Zhao , Y. S. Meng , Adv. Energy Mater. 2020, 10, 2001274.

[7] C. Vaalma , D. Buchholz , M. Weil , S. Passerini , Nat. Rev. Mater. 2018, 3, 1.

[8] F. Zheng , M. Kotobuki , S. Song , M. O. Lai , L. Lu , J. Power Sources 2018, 389, 198.

[9] Y. Wang , S. Song , C. Xu , N. Hu , J. Molenda , L. Lu , Nano Mater. Sci. 2019, 1, 91.

[10] T. Famprikis , P. Canepa , J. A. Dawson , M. S. Islam , C. Masquelier , Nat. Mater. 2019, 18, 1278. 31427742 10.1038/s41563-019-0431-3

[11] J. Wang , T. He , X. Yang , Z. Cai , Y. Wang , V. Lacivita , H. Kim , B. Ouyang , G. Ceder , Nat. Commun. 2023, 14, 5210. 37626068 10.1038/s41467-023-40669-0 PMC10457403

[12] J. B. Goodenough , H. Y.‐P. Hong , J. A. Kafalas , Mater. Res. Bull. 1976, 11, 203.

[13] R. Collongues , D. Gourier , A. Kahn , J. P. Boilot , P. Colomban , A. Wicker , J. Phys. Chem. Solids 1984, 45, 981.

[14] A. Hayashi , K. Noi , A. Sakuda , M. Tatsumisago , Nat. Commun. 2012, 3, 856. 22617296 10.1038/ncomms1843

[15] O. Maus , M. T. Agne , T. Fuchs , P. S. Till , B. Wankmiller , J. M. Gerdes , R. Sharma , M. Heere , N. Jalarvo , O. Yaffe , M. R. Hansen , W. G. Zeier , J. Am. Chem. Soc. 2023, 145, 7147. 36946557 10.1021/jacs.2c11803

[16] T. Fuchs , S. P. Culver , P. Till , W. G. Zeier , ACS Energy Lett. 2020, 5, 146.

[17] Z. Zhang , E. Ramos , F. Lalère , A. Assoud , K. Kaup , P. Hartman , L. F. Nazar , Energy Environ. Sci. 2018, 11, 87.

[18] M. A. Kraft , L. M. Gronych , T. Famprikis , S. Ohno , W. G. Zeier , Chem. Mater. 2020, 32, 6566.

[19] S. Wenzel , T. Leichtweiss , D. A. Weber , J. Sann , W. G. Zeier , J. Janek , ACS Appl. Mater. Interfaces 2016, 8, 28216. 27677413 10.1021/acsami.6b10119

[20] T. Asano , A. Sakai , S. Ouchi , M. Sakaida , A. Miyazaki , S. Hasegawa , Adv. Mater. 2018, 30, 1803075. 10.1002/adma.201803075 30216562

[21] Z. Huang , S. Yoshida , H. Akamatsu , K. Hayashi , S. Ohno , ACS Mater. Lett. 2024, 1732.

[22] J. Fu , S. Wang , D. Wu , J. Luo , C. Wang , J. Liang , X. Lin , Y. Hu , S. Zhang , F. Zhao , W. Li , M. Li , H. Duan , Y. Zhao , M. Gu , T.‐K. Sham , Y. Mo , X. Sun , Adv. Mater. 2024, 36, 2308012. 10.1002/adma.202308012 37848393

[23] H. Kwak , J. Lyoo , J. Park , Y. Han , R. Asakura , A. Remhof , C. Battaglia , H. Kim , S.‐T. Hong , Y. S. Jung , Energy Storage Mater. 2021, 37, 47.

[24] R. Schlem , A. Banik , M. Eckardt , M. Zobel , W. G. Zeier , ACS Appl. Energy Mater. 2020, 3, 10164.

[25] Y. Qie , S. Wang , S. Fu , H. Xie , Q. Sun , P. Jena , J. Phys. Chem. Lett. 2020, 11, 3376. 32282213 10.1021/acs.jpclett.0c00010

[26] D. Park , K. Kim , G. H. Chun , B. C. Wood , J. H. Shim , S. Yu , J. Mater. Chem. A. 2021, 9, 23037.

[27] J. Park , J. P. Son , W. Ko , J.‐S. Kim , Y. Choi , H. Kim , H. Kwak , D.‐H. Seo , J. Kim , Y. S. Jung , ACS Energy Lett. 2022, 7, 3293.

[28] Z. Wei , L. F. Nazar , J. Janek , Batter. Supercaps, 202400005.

[29] S. Ohno , A. Banik , G. F. Dewald , M. A. Kraft , T. Krauskopf , N. Minafra , P. Till , M. Weiss , W. G. Zeier , Prog. Energy 2020, 2, 022001.

[30] E. A. Wu , S. Banerjee , H. Tang , P. M. Richardson , J.‐M. Doux , J. Qi , Z. Zhu , A. Grenier , Y. Li , E. Zhao , G. Deysher , E. Sebti , H. Nguyen , R. Stephens , G. Verbist , K. W. Chapman , R. J. Clément , A. Banerjee , Y. S. Meng , S. P. Ong , Nat. Commun. 2021, 12, 1256. 33623048 10.1038/s41467-021-21488-7 PMC7902639

[31] E. Sebti , J. Qi , P. M. Richardson , P. Ridley , E. A. Wu , S. Banerjee , R. Giovine , A. Cronk , S.‐Y. Ham , Y. S. Meng , S. P. Ong , R. J. Clément , J. Mater. Chem. A 2022, 10, 21565.

[32] P. Ridley , L. H. B. Nguyen , E. Sebti , B. Han , G. Duong , Y.‐T. Chen , B. Sayahpour , A. Cronk , G. Deysher , S.‐Y. Ham , J. A. S. Oh , E. A. Wu , D. H. S. Tan , J.‐M. Doux , R. Clément , J. Jang , Y. S. A. Meng , Matter 2024, 7, 485.

[33] T. Zhao , A. N. Sobolev , R. Schlem , B. Helm , M. A. Kraft , W. G. Zeier , ACS Appl. Energy Mater. 2023, 6, 4334.

[34] T. Zhao , A. N. Sobolev , X. M. I. Labalde , M. A. Kraft , W. G. Zeier , J. Mater. Chem. A 2024, 12, 7015.

[35] T. Dai , S. Wu , Y. Lu , Y. Yang , Y. Liu , C. Chang , X. Rong , R. Xiao , J. Zhao , Y. Liu , W. Wang , L. Chen , Y.‐S. Hu , Nat. Energy 2023, 8, 1221.

[36] L. Zhou , J. D. Bazak , C. Li , L. F. Nazar , ACS Energy Lett. 2024, 9, 4093.

[37] T. Zhao , B. Samanta , X. M. De Irujo‐Labalde , G. Whang , N. Yadav , M. A. Kraft , P. Adelhelm , M. R. Hansen , W. G. Zeier , ACS Mater. Lett. 2024, 6, 3683.

[38] X. Lin , S. Zhang , M. Yang , B. Xiao , Y. Zhao , J. Luo , J. Fu , C. Wang , X. Li , W. Li , F. Yang , H. Duan , J. Liang , B. Fu , H. Abdolvand , J. Guo , G. King , X. Sun , Nat. Mater. 2025, 24, 83. 39354087 10.1038/s41563-024-02011-x PMC11685097

[39] X. Lin , Y. Zhao , C. Wang , J. Luo , J. Fu , B. Xiao , Y. Gao , W. Li , S. Zhang , J. Xu , F. Yang , X. Hao , H. Duan , Y. Sun , J. Guo , Y. Huang , X. Sun , Angew. Chem. 2024, 136, 202314181. 10.1002/anie.202314181 38009453

[40] S. Zhang , Y. Xu , H. Wu , T. Pang , N. Zhang , C. Zhao , J. Yue , J. Fu , S. Xia , X. Zhu , G. Wang , H. Duan , B. Xiao , T. Mei , J. Liang , X. Sun , X. Li , Angew. Chem., Int. Ed. 2024, 63, 202401373. 10.1002/anie.202401373 38659181

[41] S. Kmiec , E. Ruoff , A. Manthiram , Angew. Chem., Int. Ed. 2025, 64, 202416979. 10.1002/anie.202416979 39347887

[42] E. Ruoff , S. Kmiec , A. Manthiram , Adv. Energy Mater. 2024, 14, 2402091.

[43] E. Ruoff , S. Kmiec , A. Manthiram , ACS Appl. Mater. Interfaces 2025, 17, 18420. 40094335 10.1021/acsami.5c00755

[44] J. L. Sudworth , J. Power Sources 2001, 100, 149.

[45] M. H. Levitt , Spin Dynamics: Basics of Nuclear Magnetic Resonance, John Wiley & Sons, Chichester, England 2008.

[46] J. T. S. Irvine , D. C. Sinclair , A. R. E. West , Adv. Mater. 1990, 2, 132.

[47] A. Padhi , K. S. Nanjundaswamy , J. Goodenough , J. Electrochem. Soc. 1997, 144, 1188.

[48] D. Morgan , A. V. Ven , G. L. Ceder , Electrochem. Solid‐State Lett. 2003, 7, A30.

[49] S. Nishimura , G. Kobayashi , K. Ohoyama , R. Kanno , M. Yashima , A. Yamada , Nat. Mater. 2008, 7, 707. 18690238 10.1038/nmat2251

[50] R. Malik , A. Abdellahi , G. Ceder , J. Electrochem. Soc. 2013, 160, A3179.

[51] M. Häfner , M. Bianchini , J. Phys. Chem. C. 2024, 128, 19978. 10.1021/acs.jpcc.4c05559 PMC11613591 39634025

[52] Y. Hu , J. Fu , J. Xu , J. Luo , F. Zhao , H. Su , Y. Liu , X. Lin , W. Li , J. T. Kim , X. Hao , X. Yao , Y. Sun , J. Ma , H. Ren , M. Yang , Y. Huang , X. Sun , Matter 2024, 7, 1018.

[53] L. Hu , H. Li , F. Chen , Y. Liu , J. Wang , C. Ma , J. Energy Chem. 2024, 95, 1.

[54] M. Wu , X. Liu , H. Liu , D. Li , X. Qi , J. Zeng , L. Gao , C.‐W. Nan , L.‐Z. Fan , Nat. Commun. 2025, 16, 2808. 40118844 10.1038/s41467-025-58113-w PMC11928663

