# Supplementary material for: Structure and Ionic Conductivity of Halide Solid Electrolytes Based on NaAlCl4 and Na2ZnCl4
Source: Adv Sci (Weinh). 2025 May 28;12(30):e07224. doi: 10.1002/advs.202507224 (PMC12376589; doi:10.1002/advs.202507224)
Supplement: Supplementary file 1 — Supporting Information [file ADVS-12-e07224-s001.pdf]

ADVANCED SCIENCE
Open Access

Supporting Information

for *Adv. Sci.*, DOI 10.1002/advs.202507224

Structure and Ionic Conductivity of Halide Solid Electrolytes Based on $NaAlCl_4$ and Na_2ZnCl_4

Hao Guo, Michael Häfner, Helen Grüninger and Matteo Bianchini**

SUPPLEMENTARY INFORMATION

Structure and Ionic Conductivity of Halide Solid Electrolytes based on $NaAlCl_4$ and Na_2ZnCl_4

Hao Guo,[ab] Michael Häfner,[ab] Helen Grüninger,[ab*] Matteo Bianchini[ab*]

[a] *Department of Chemistry, University of Bayreuth, Universitätstraße 30, 95447 Bayreuth, Germany*

[b] *Bavarian Center for Battery Technology (BayBatt), Weiherstraße 26, 95448 Bayreuth, Germany*

*Corresponding authors: matteo.bianchini@uni-bayreuth.de, helen.grueninger@uni-bayreuth.de

Experimental Methods

Synthesis. A series of compositions with the nominal formula $Na_{1+x}Zn_xAl_{1-x}Cl_4$ was synthesized by the mechanochemical method. Stoichiometric amounts of NaCl (Thermo Fisher, 99.5% purity, pre-dried at 60 °C for 3 days under 50 mbar), $AlCl_3$ (Sigma Aldrich, 99% purity), and $ZnCl_2$ (Thermo Fisher, 99.995% purity) were ground with mortar and pestle by hand for 10 minutes to guarantee a homogeneous mixing process. The mixture of precursors was sealed in a zirconia jar (45 mL, Fritsch GmbH), and the mass ratio between milling media (ZrO_2, 5 mm diameter) and precursors was 36:1. A Pulverisette 7 planetary ball milling machine (Fritsch GmbH) was set at 100 rpm for 10 min to pre-mix the precursors. Afterward, ball milling was carried out for 96 cycles at 600 rpm, each cycle including 5 minutes of ball milling and then a 10-minute break to avoid overheating.

For the preparation of anode Na_3Sn, stoichiometric amounts of Na metal (Sigma Aldrich, 99.9%) and Sn metal (nanopowder, US Research Nanomaterials, 99.9%) were prepared by

grinding in mortar for 30 minutes, followed by ball milling in a zirconia jar (45 mL, Fritsch GmbH), keeping mass ratio between milling media (ZrO_2, 5 mm diameter) and precursors at 36:1. A pre-mixing process was operated at 100 rpm for 10 minutes, followed by 8 h ball-milling at 300 rpm. The synthesized Na_3Sn has a potential of ~ 0.19 V vs. Na^+/Na.

For the preparation of the solid-state electrolyte Na_3PS_4 interlayer a stoichiometric mixture of Na_2S (Thermo scientific, 99%) and P_2S_5 (Sigma Aldrich, 99%) was prepared by ball milling with a molar ratio of 3:1. The mixture was transferred into quartz ampoules in an Ar-filled glovebox and then sealed with an acetylene torch under vacuum, followed by a heat treatment at 270 °C for 3 h in a furnace. After annealing, the powder was naturally cooled down and hand-ground for further characterization and cell fabrication.

The $NaCrO_2$ cathode material was synthesized by mixing a stoichiometric amount of Na_2CO_3 (Thermo Scientific, 99.95%), Cr_2O_3 (Sigma Aldrich, >98%), and the mixture was mixed thoroughly by ball-milling (SPEX 8000D). Then, the mixture was pressed into pellets and annealed in a tube furnace under Argon. The furnace was heated to 900 ℃ with a ramping rate of 5 ℃/min and kept at 900 ℃ for 10h. Afterwards, the furnace was cooled down naturally to room temperature.

All the synthesis work above was performed, and all synthesized products were transferred to, an argon-filled glovebox (200 B, MBraun, Germany; H_2O < 0.1 ppm, O_2 < 0.1 ppm). Ball milling jars are sealed and opened under the same argon atmosphere.

X-ray diffraction and Rietveld refinement. XRD patterns were collected on a Laboratory STOE STADI P diffractometer (Mo Kα_1, λ = 0.7093 Å, Mythen 2K detector, tube operated at 50 kV and 40 mA) in Debye-Scherrer scan mode. All samples were sealed in 0.5 mm diameter glass capillaries (Hilgenberg GmbH). Data collection was carried out for all samples in 0.015° steps from 2.222° to 75.587°. For the synchrotron XRD (sXRD), analysis was carried out in transmission mode at NOTOS beamline at ALBA Synchrotron Light Source, Barcelona, Spain. The synchrotron light was first vertically collimated, then monochromatized using two pairs of liquid-cooled Si (111) crystals and finally focused on the sample position down to an ∼ 800 × 500 μm^2 beam spot. XRD patterns were acquired at 13 keV (0.9537 Å) in the 2θ range of 7–50° for 30 s using a Mythen detector.

The Rietveld refinement of structural models versus the XRD patterns was conducted by the Fullprof Suite. Rietveld refinements consisted of the following steps: (1) background selection,

zero-point refinements (2) lattice parameters refinements, (3) scale factor refinement, (4) peak shape parameters refinements (U, X, and Y) from the Thompson−Cox−Hastings pseudo-Voigt function, (5) site fractional coordinates and isotropic displacement parameters refinements, (6) site occupancy refinements. All the unit cell parameters and detailed information can be found in Tables S1-S13.

Electrochemical Impedance Spectroscopy. Electrochemical impedance spectroscopy (EIS) was used to study the ionic conductivities and activation energy of the samples. Sample powder was filled in a 12 mm diameter polyether ether ketone (PEEK sleeve with an architecture {hard metal |SSE| hard metal} symmetric cell and cold-pressed pellets were prepared under an applied pressure of 430 MPa for 1 h in a CompreDrive instrument from rhd instruments GmbH. The hard metal is tungsten carbide, which functions as Na^+ blocking layer. The impedance measurements were carried out using an SP-200 potentiostat (Biologic) applying an AC amplitude of 100 μA (GEIS) and frequencies in the range of 200 kHz to 100 mHz. Spectra were collected under different pressures (2 MPa to 400 MPa) and at different temperatures of 5 to 55 °C. To obtain reliable fitting results, the ultrahigh frequency range was discarded due to the inductor influence from instrumental interconnection. Activation energies were derived from a linearized Arrhenius plot: $\ln(\sigma T) \propto 1/T$.

Electrochemical characterization. The DC polarization method was applied to determine the electronic conductivity by applying different constant bias voltages over 600 s and obtaining the corresponding steady-state current. Linear sweep voltammetry (LSV) was conducted at 0.1 mV/s to investigate the electrochemical stability using the following cell configuration: Na_3Sn|NPS|chloride SSE composite, the mass ratio of each layer was 80 mg: 100 mg: 120 mg, respectively. Each sample powder was pressed in a 12 mm diameter PEEK sleeve at 300 MPa for 10 min. The chloride SSE composite consisted of 83.3 wt. % chloride SSE and 16.7 wt. % carbon SuperP, the latter ensuring electronic transfer within the composite. DC polarization and LSV measurements are performed under external pressure of 70 MPa and at room temperature.

All-solid-state full cells were assembled using NZAC (*x* = 0.5) SSE in combination with $NaCrO_2$ ($q_{theo.}$ = 120 mAh/g) as cathode, Na_3PS_4 as interlayer, and Na_3Sn alloy as anode in an argon-filled glovebox. The cathode composite was prepared by mixing $NaCrO_2$, NZAC SSE and carbon SuperP in an agate mortar by hand, the weight ratio of these 3 materials is 47.4: 47.4: 5.2. For the cell assembly, firstly 100 mg of Na_3PS_4 powder was placed into the PEEK sleeve (12 mm

diameter) and pressed at 250 MPa for 5 min. Then, 50 mg of Na_3Sn were spread evenly on one side of the Na_3PS_4 and further pressed at 250 MPa for 5 mins. Finally, 19 mg of the cathode composite was spread evenly on the other side of Na_3PS_4 pellet and pressed at 250 MPa for 2 min. The cells were cycled under an external pressure of 70 MPa, and the galvanostatic charge-discharge measurement at 0.1 C was conducted on an SP-200 potentiostat (Biologic) with a potential range of 1.8 ~ 3.5 V versus Na_3Sn.

Nuclear magnetic resonance measurements. Solid-state NMR experiments were performed using a Bruker Avance II spectrometer operating at a magnetic field strength of 9.4 T, corresponding to a ^{1}H Larmor frequency of 400 MHz and ^{23}Na Larmor frequency of 105.8 MHz. All experiments were carried out using a Bruker 4.0 mm H-X MAS NMR probe at a MAS frequency of 12.5 kHz unless otherwise stated. All ^{23}Na spectra were referenced using a 0.1M NaCl solution (aq) at room temperature.

1D ^{23}Na MAS single-pulse (SP) NMR spectra were recorded after a selective 90° pulse of 10.5 µs (B_1 field strength ~ 23.8 kHz) to excite only the central transition. The recycle delays were optimized for each sample to ensure quantitative NMR spectra. 2D ^{23}Na MQMAS NMR spectra were recorded using a *z*-filtered 4-pulse sequence [1] with a split-t_1 evolution at a magnetic field of 9.4 T (MAS rate 12.5 kHz, recycle delay optimized per sample, selective 90° pulse at B_1-field of 24-27 kHz, z filter 20µs) to extract isotropic chemical shifts and average quadrupolar coupling constants. These were then used to deconvolute the 1D ^{23}Na MAS NMR spectra using the software ssNake.[2]

2D ^{23}Na-^{23}Na exchange (EXSY) correlation spectra were acquired using a ^{23}Na B_1 field strength of ~45 kHz and a mixing time of 3 ms. A rotor-synchronized t_1 evolution (160µs) was employed between two ^{23}Na pulses.

Computational methods. All density functional theory calculations (DFT) were carried out with the plane-wave program package VASP[3–5] and PAW pseudopotentials[6] with the generalized-gradient functional PBE[7] at a cutoff of 520 eV. The cutoff was chosen based on the parameters for the materials project.[8] The dispersion was accounted for with the DFT-D3 dispersion correction by Grimme *et al.* with Becke-Johnson damping.[9,10] The calculation workflow was coordinated using pymatgen[11] and ASE.[12] The k-point grid for the structures was generated at a grid density of 600 per number of atoms. The convergence criteria for DFT structure optimizations were set to a threshold of 0.01 eV/Å for the highest normed atomic force and 1×10^{-5} eV for the energy in the self-consistent field (SCF) calculation. In addition, the

initial configuration analysis and the molecular dynamics calculations were carried out with the MACE-MP-0 machine-learned foundational model,[13] which is based on MACE,[14,15] with a PFP-based pyTorch implementation of DFT-D3.[16] The configurational analysis was carried out with the program supercell,[17] which generates all possible permutations for a given list of atom positions and occupancies. The number of generated structures was usually limited to a subset of *N* configurations with the lowest Coulomb energy because of the high number of possible permutations, where *N* is between 10 and 100.

The molecular dynamics calculations were carried out using an NPT ensemble with combined Nosé-Hoover and Parrinello-Rahman dynamics for the thermostat[18–20] at a standard pressure of 1.01325 bar, characteristic times of 25 fs for ttime and 75 fs for ptime, and a time step of 1.5 fs. Initial equilibration was done for 5000 steps or 7.5 ps, and the production runs were run for 50000 steps or 75 ps. A few additional runs were carried out for 250000 steps, representing an MD simulation time of 350 ps to confirm that 75 ps simulation time is sufficient for simulating Na diffusion in the given models (Figure S10f). The supercells for the simulation were set to have lattice vector lengths of at least 24 Å, which yields models with about 650 atoms. Nudged elastic band calculations were carried out in VASP using the code by Henkelman *et al.* [21–26] and in ASE[21–23,27,28] with a force convergence criterion at 0.01 eV/Å and climbing image convention. For the NMR calculations, the cutoff energy was raised to 600 eV and the energy convergence for the SCF to 1×10^{-10} eV to ensure the accuracy of the linear response calculations for the chemical shifts. The nuclear quadrupole moments required for the interpretation of the results are based on the review by Pyykkö.[29] Existing structure information was sourced from the online structure database ICSD.[30] All structure images were generated with VESTA (version 3.5.8)[31]and all diagrams were created using matplotlib.[32]

Figures

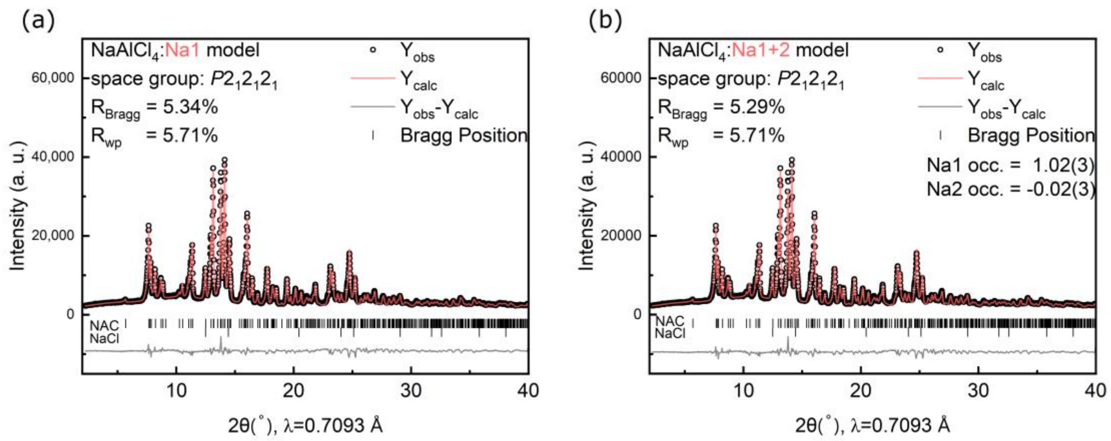

Figure S1. (a) Rietveld refinement of $NaAlCl_4$ based on Na1 model. (b) Rietveld refinement of NAC based on Na1+2 model.

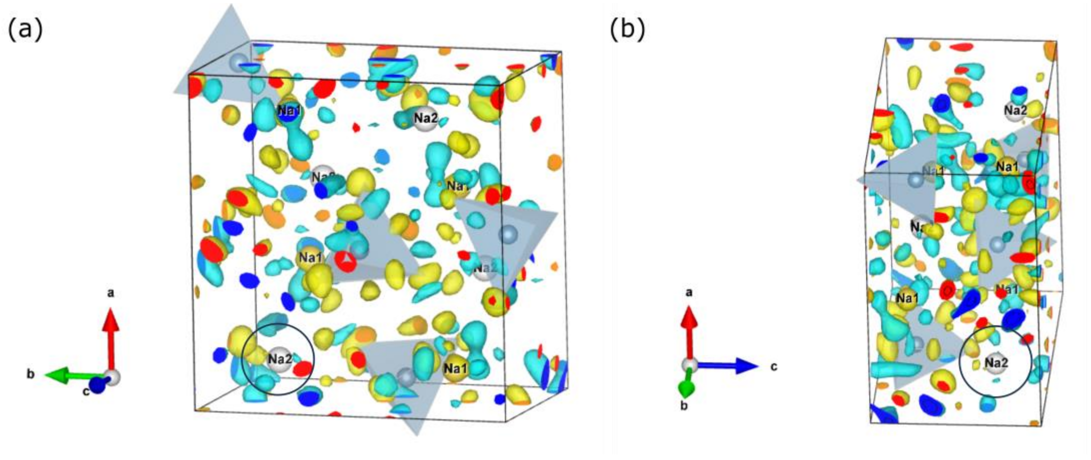

Figure S2. (a, b) Fourier difference maps derived from Rietveld refinement are shown in different views of points. The potential Na2 site (grey sphere) is emphasized in a black circle.

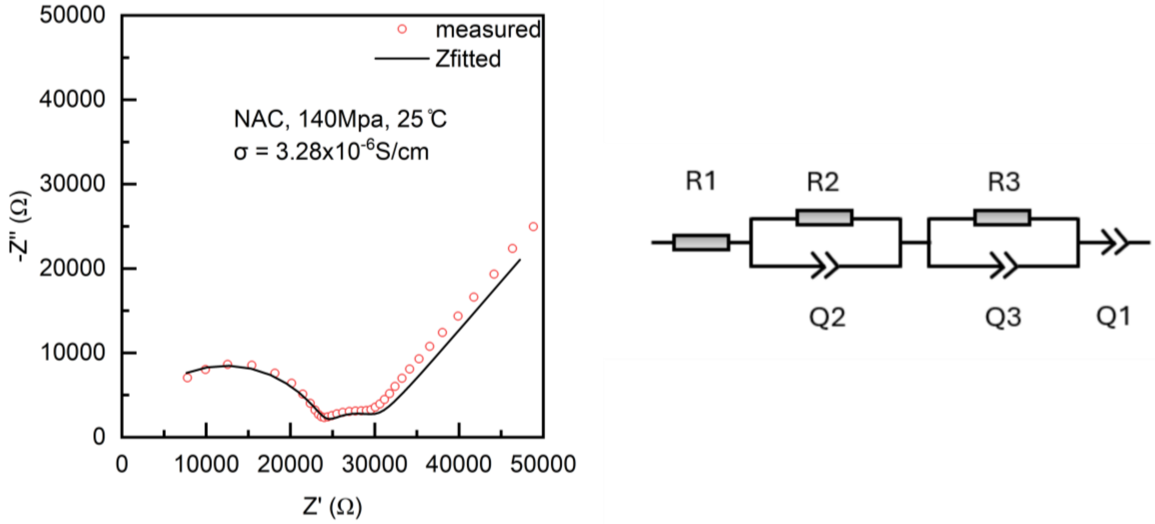

Fitting Parameters	R1	R2	Q2	a2	R3	Q3	a3	Q1	a1
Value	6.36e-5	24383	1.8e-9	0.7695	4711	0.5e-6	0.834	20.06e-6	0.54
Unit	Ω	Ω	F*s^(a-1)		Ω	F*s^(a-1)		F*s^(a-1)	

Results of equivalent circuit fitting.

Figure S3. Nyquist plot and corresponding equivalent circuit fitting of ball-milled NAC sample.

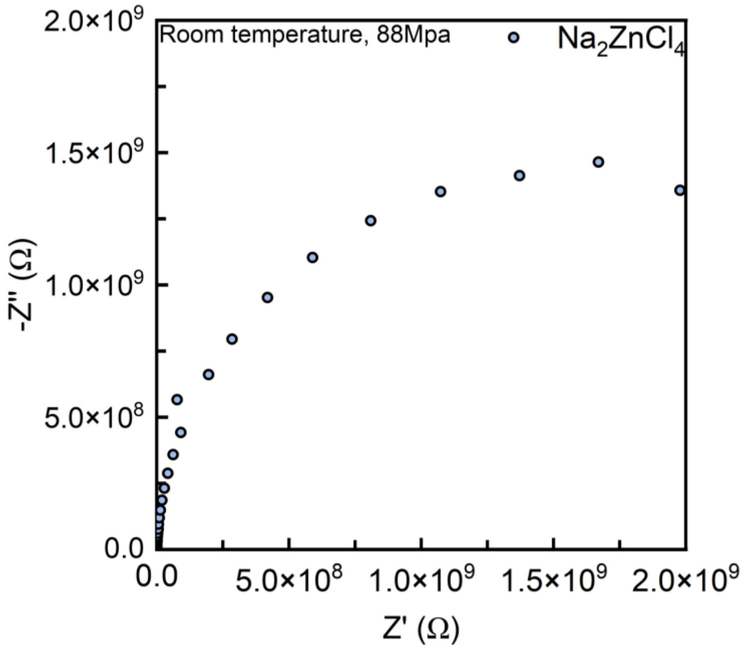

Figure S4. Representative Nyquist plot of NZC pristine material at room temperature.

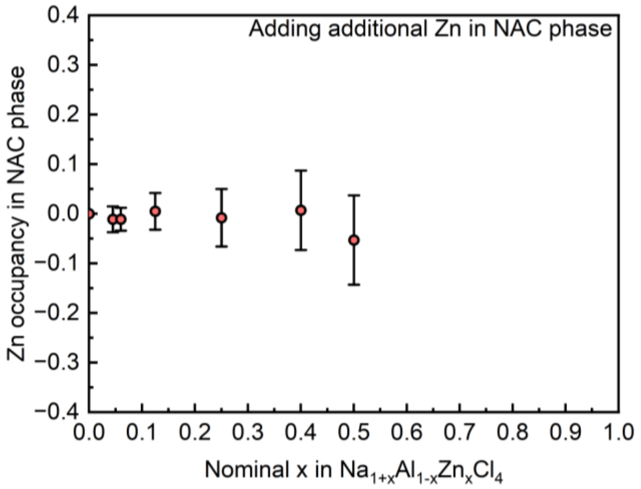

Figure S5. Refined Zn^{2+} occupancy in the NAC phase.

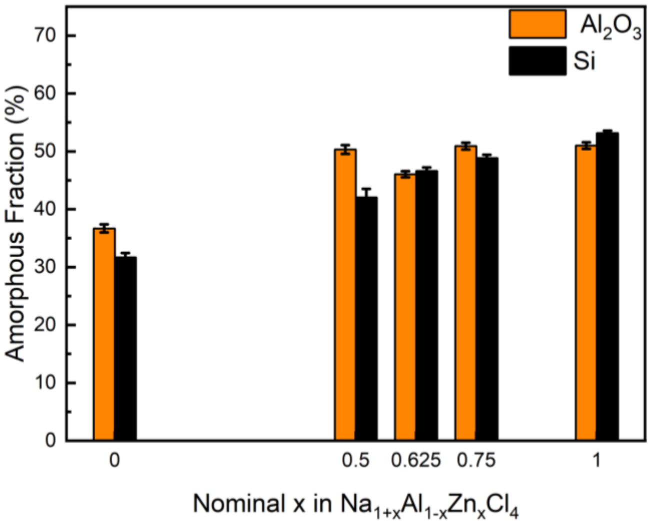

Figure S6. Amorphous fraction of NZAC compositions. Calculations based on the Rietveld refinement of the internal reference Si and Al_2O_3. Because our samples have a similar crystallographic density to Si, leading to more homogeneous capillary filling, we deem the Si values more trustworthy (Al_2O_3, on the other hand, is denser).

(a)

(b)

(c)

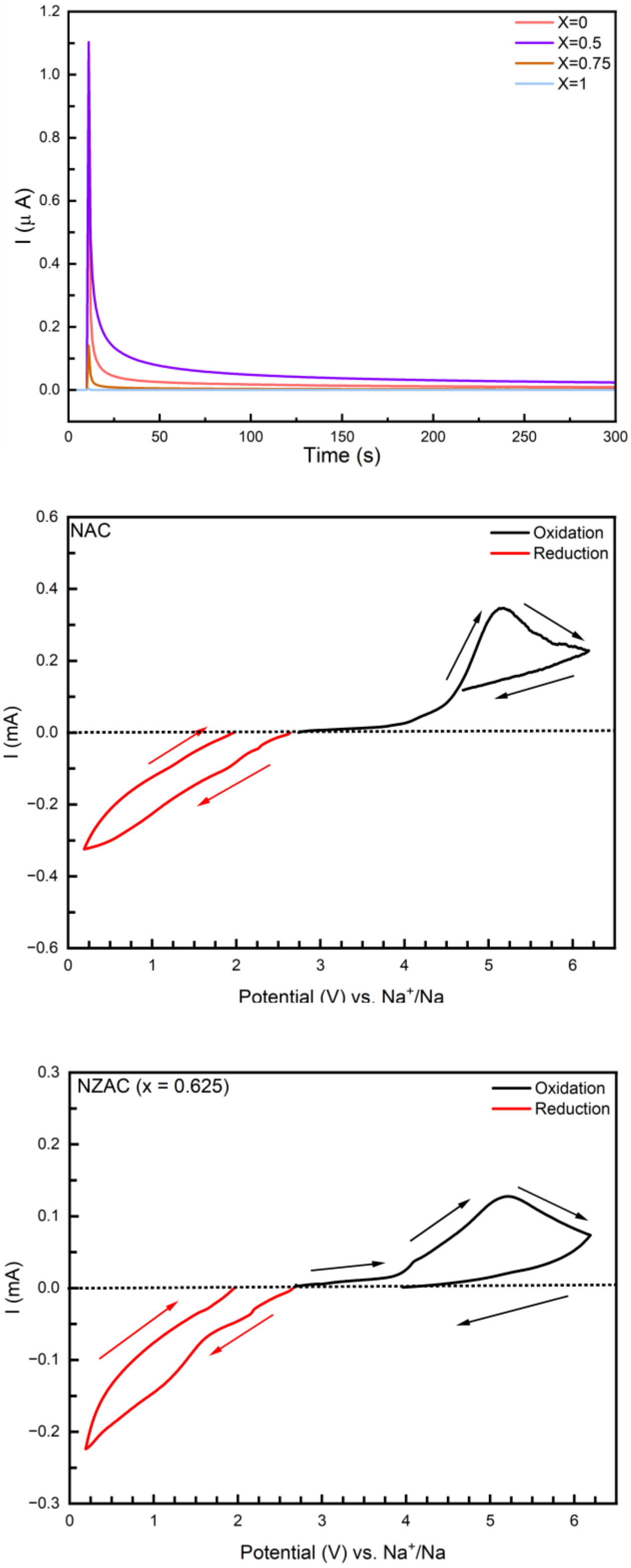

Figure S7. (a) DC polarization measurements of NZAC composition *x* = 0, 0.5, 0.75, 1. First cycle of linear sweep voltammetry scan at 0.1 mV/s for (b) NAC and (c) NZAC (*x* = 0.625). Positive and negative sweeps were measured in 2 separate cells.

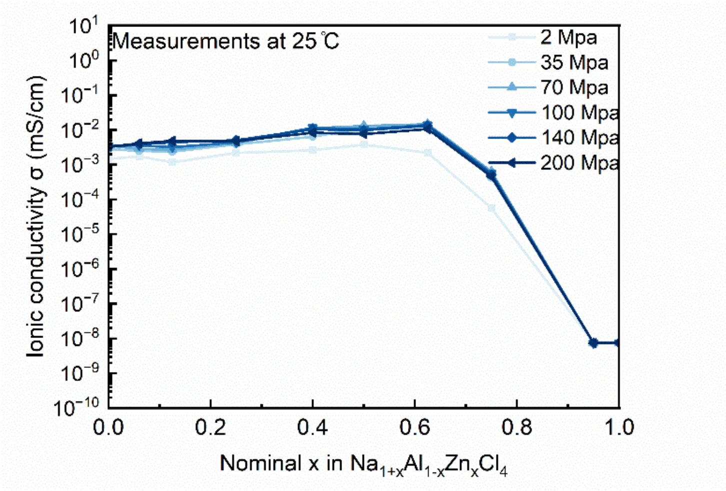

Figure S8. Ionic conductivities (logarithmic scale) of $Na_{1+x}Zn_xAl_{1-x}Cl_4$ at 25 °C as a function of applied pressure.

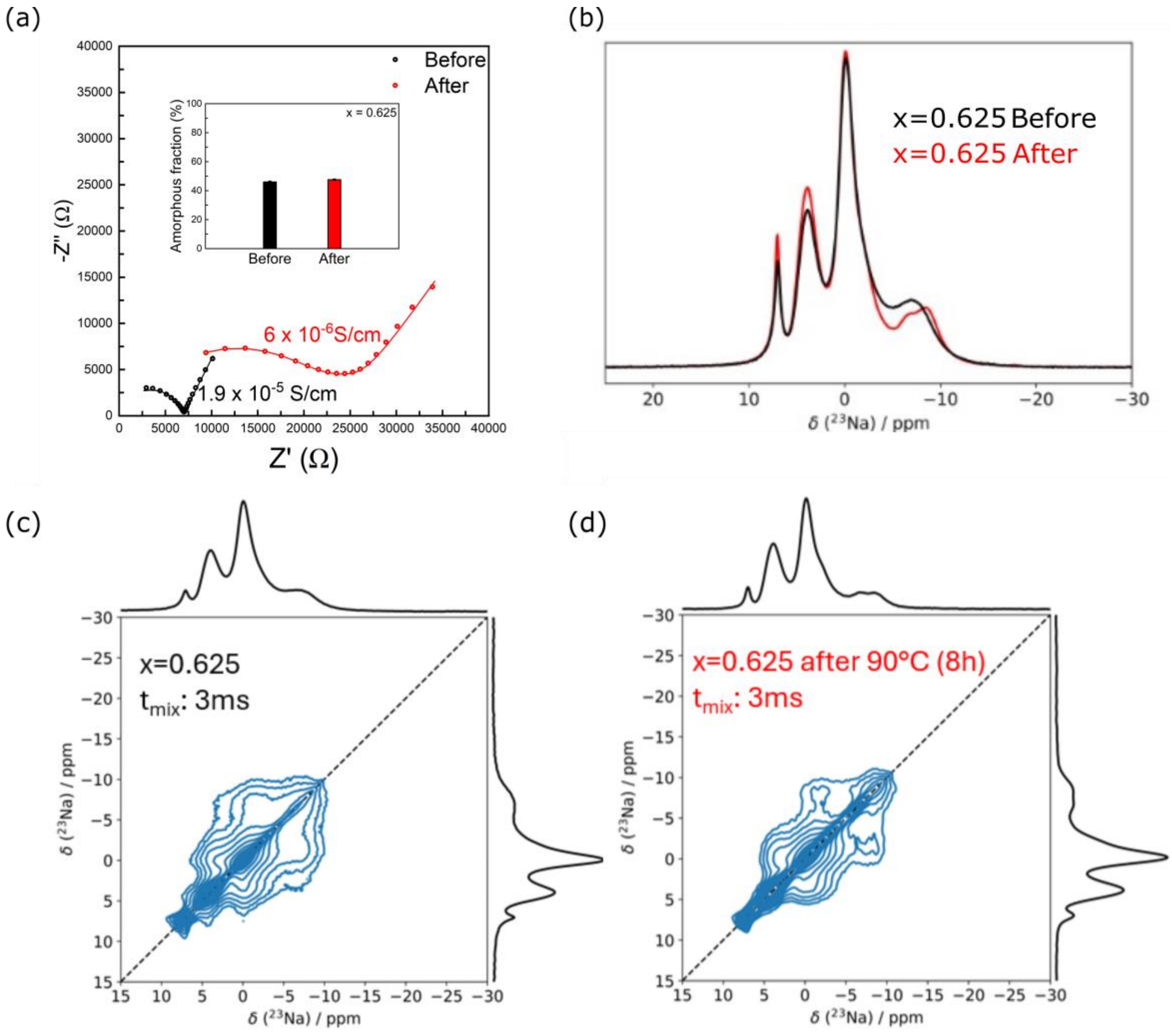

Figure S9. Evolutions of NZAC composition x = 0.625 before and after annealing at 90 °C for 8 hours. (a) Nyquist Plot and ionic conductivity evolution, amorphous fraction evolution as inset. (b) 1D ^{23}Na NMR spectra evolution; 2D ^{23}Na-^{23}Na EXSY NMR spectra (c) before and (d) after annealing.

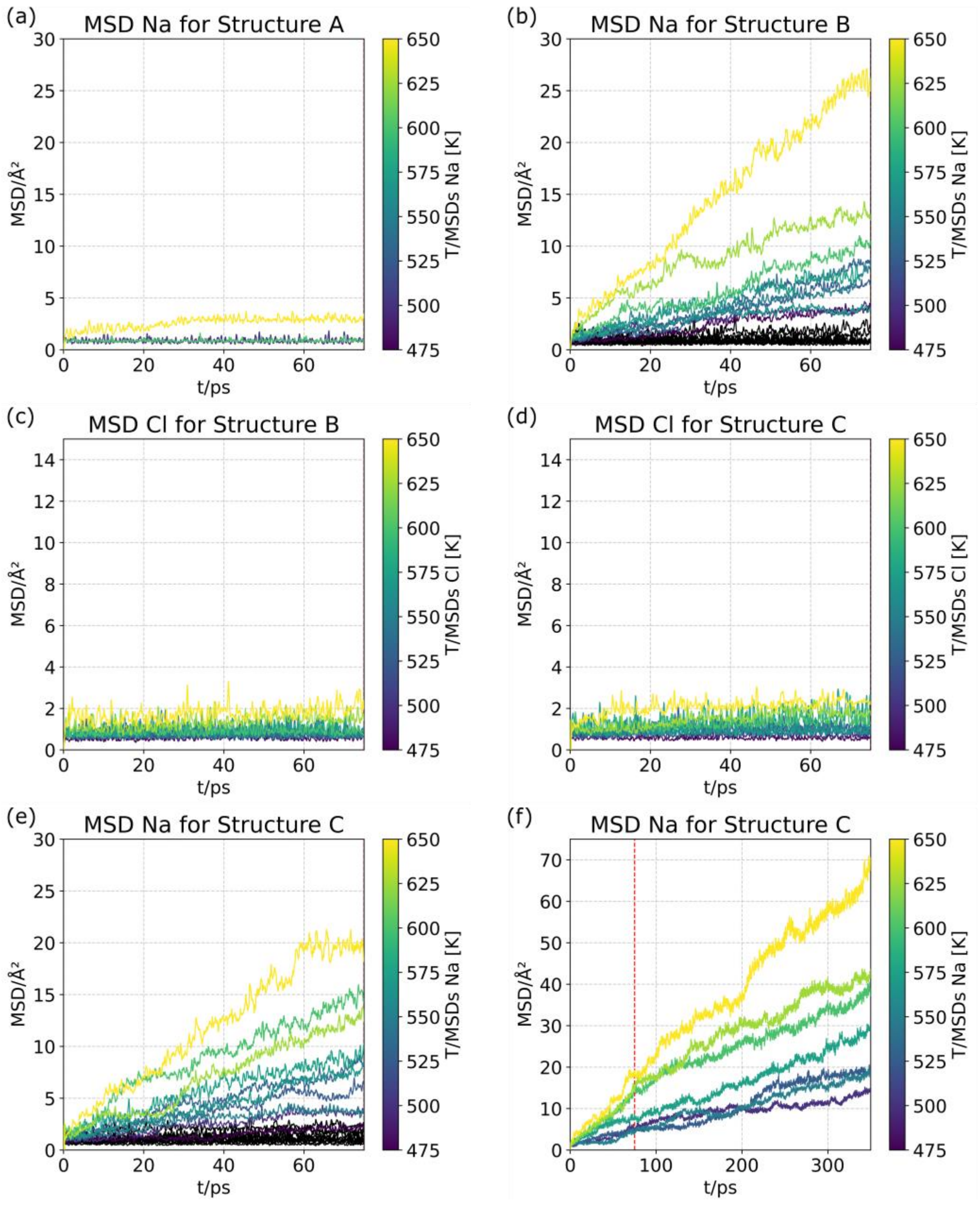

Figure S10. MSDs of the MD simulations of structures A, B, and C. (a) MSDs of Na in structure A. (b) MSDs of Na in structure B. (c) MSDs of Cl in structure B. (d) MSDs of Cl in structure C. (e) MSDs of Na in structure C. (f) MSDs of Na in structure C for 350 ps MD simulations. The dashed red line indicates the standard simulation length of the other MD runs.

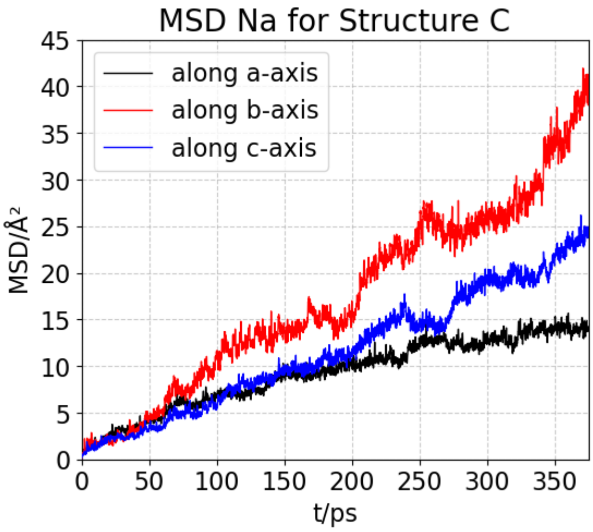

Figure S11. 1D-MSDs of Na in the MD simulations of structure C at 600 K, separated by movement along the crystal lattice axes *a*, *b*, and *c* as shown in Figures 9d and 9e.

To evaluate the deviation of MACE-MP-0/D3(BJ) from PBE/D3(BJ), 50000-step MD simulations of the structures A, B, and C were performed at 600 K with a supercell of 2x2x1 containing ~200 atoms. A smaller supercell was used than for the production runs so that the DFT calculations for it could be performed at a more reasonable calculation speed. For the comparison, a DFT single point calculation was performed on every 2000th step of the simulation, the resulting errors are shown in Figure S12 below.

The force errors are substantially larger for the NPT-Ensemble MD simulations, with an RMSD between 70 and 120 meV/Å, indicating that MACE-MP-0 is worse at describing systems at an elevated temperature. The energy is consistently overestimated by MACE-MP-0, but the error is similar for all systems.

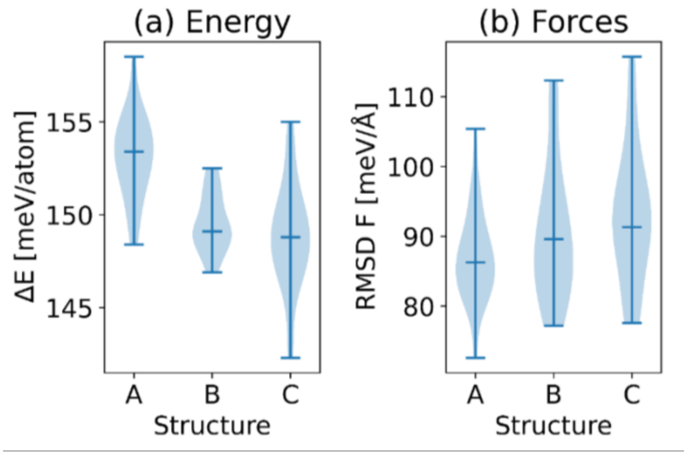

Figure S12. Deviation of the MACE-MP-0/D3(BJ) results from the PBE/D3(BJ) results obtained with VASP. (a) Deviation of the energy. (b) RMSD of the Forces.

NEB Pathways for $Na_{1.75}Zn_{0.75}Al_{0.25}Cl_4$

The NEB calculations for structure **A** were also carried out on PBE/D3(BJ) in VASP to check how much MACE-MP-0/D3(BJ) deviates from pure ab initio results. Due to the computational cost of DFT calculations, only a 1x1x2 supercell of the structure in Figure 8a was employed. The result is shown in Figure S13:

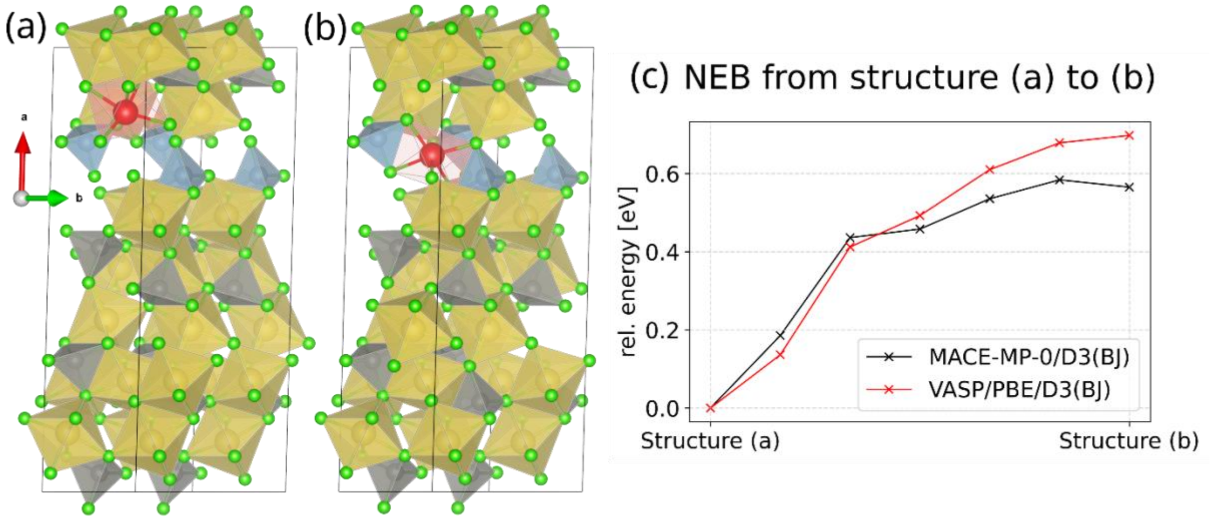

Figure S13. (a) Starting structure for the NEB. (b) End structure for the NEB. (c) NEB results for MACE-MP-0/D3(BJ) and VASP/PBE/D3(BJ). The activation energies deviate by 0.12 eV, mostly due to MACE-MP-0/D3(BJ) underestimating the stability of structure S13b.

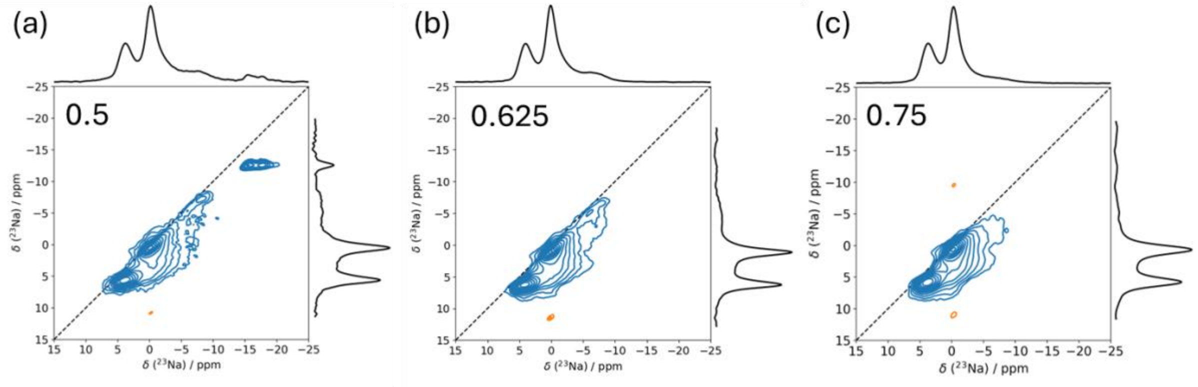

Figure S14. 2D ^{23}Na MQMAS NMR spectra (z-filtered) of NZAC compositions (a) $x = 0.5$, (b) $x = 0.625$, and (c) $x = 0.75$.

DFT calculations of NMR parameters

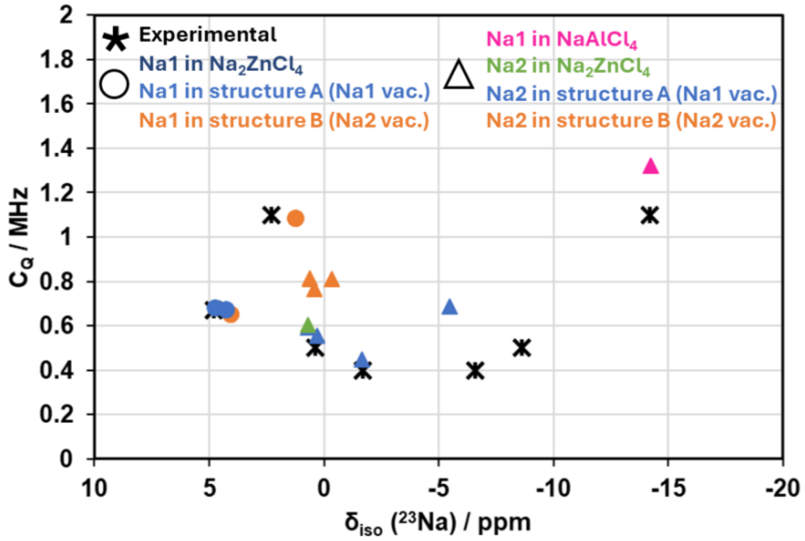

Figure S15. ^{23}Na NMR parameters (isotropic chemical shift and δ_{iso} and quadrupolar coupling C_Q) derived from DFT calculations of $NaAlCl_4$ (pink), Na_2ZnCl_4 (green), and $Na_{1.75}Zn_{0.75}Al_{0.25}Cl_4$ with solely Na1 vac. (blue) or Na2 vac. (orange). The circles denote Na1 sites, while the triangles denote Na2 sites. For comparison, the experimentally observed NMR parameters of the different sites are depicted as black stars.

Tables

Table S1: Structural parameters obtained from Rietveld refinement of $NaAlCl_4$. a) Model with only Na1 site occupied; b) model with only Na2 site occupied; c) model with both sites can be occupied.

**a) NAC (Na 1 model)**						
S.G.: *P* $2_12_12_1$ (19); Z = 4						R_{Bragg} = 5.34%
a = 10.349(1) Å; *b* = 9.900(1) Å; *c* = 6.177(1) Å; *V* = 632.863(135) Å^3; *V/Z* = 158.216(34) Å^3						R_{wp} = 5.71%
						χ^2 = 16.0
		atomic position				
atoms	Wyckoff position	*x/a*	*y/b*	*z/c*	Occ.	B_{iso}
Al	4 a	0.037(2)	0.479(3)	0.200(3)	1	1.4(6)
Cl1	4 a	0.031(2)	0.500(3)	0.548(2)	1	1.9(2)
Cl2	4 a	0.152(2)	0.316(2)	0.110(3)	1	1.9(2)
Cl3	4 a	0.348(1)	0.026(2)	0.928(3)	1	1.9(2)
Cl4	4 a	0.381(2)	0.334(2)	0.577(4)	1	1.9(2)
Na1	4 a	0.119(3)	0.219(3)	0.702(5)	1	3.0(9)

**b) NAC (Na 2 model)**						
S.G.: *P* $2_12_12_1$ (19); Z = 4						R_{Bragg} = 9.60%
a = 10.348(2) Å; *b* = 9.900(2) Å; *c* = 6.178(1) Å; *V* = 632.870(245) Å^3; *V/Z* = 158.218(61) Å^3						R_{wp} = 8.57%
						χ^2 = 36.20
		atomic position				
atoms	Wyckoff position	*x/a*	*y/b*	*z/c*	Occ.	B_{iso}
Al	4 a	0.033(4)	0.500(7)	0.207(6)	1	2(1)
Cl1	4 a	0.033(3)	0.484(5)	0.553(5)	1	1.6(3)
Cl2	4 a	0.145(4)	0.311(4)	0.072(7)	1	1.6(3)
Cl3	4 a	0.346(3)	0.023(4)	0.928(6)	1	1.6(3)
Cl4	4 a	0.374(4)	0.345(4)	0.620(6)	1	1.6(3)
Na2	4 a	0.373(7)	0.217(7)	0.21(1)	1	6(2)

c) NAC (Na 1+2 model)						
S.G.: $P\ 2_12_12_1$ (19); Z = 4						R_{Bragg} = 5.29%
a = 10.349(1) Å; b = 9.900(1) Å; c = 6.177(1) Å; V = 632.844(135) Å^3; V/Z = 158.211(34) Å^3						R_{wp} = 5.71%
						χ^2 = 15.90
		atomic position				
atoms	Wyckoff position	*x/a*	*y/b*	*z/c*	Occ.	B_{iso}
Al	4 a	0.03609	0.47886	0.20057	1	1.5(5)
Cl1	4 a	0.03097	0.50029	0.54697	1	1.8(2)
Cl2	4 a	0.15151	0.31668	0.1111	1	1.8(2)
Cl3	4 a	0.34785	0.02636	0.92783	1	1.8(2)
Cl4	4 a	0.38113	0.33384	0.57699	1	1.8(2)
Na1	4 a	0.11975	0.21934	0.70243	1.02(3)	3.4(9)
Na2	4 a	0.37013	0.21727	0.21156	-0.02(3)	3.4(9)

- Na1 + Na2 total occupancy is set to 1

Table S2: Optimized structural parameters obtained from Rietveld refinement of $NaAlCl_4$ based on synchrotron XRD data (sXRD).

NAC (Na 1 model)						
S.G.: $P\ 2_12_12_1$ (19); Z = 4						R_{Bragg} = 5.41%
a = 10.338(1) Å; b = 9.892(1) Å; c = 6.171(1) Å; V = 631.057(67) Å^3; V/Z = 157.764(17) Å^3						R_{wp} = 3.71%
						χ^2 = 20.7
		atomic position				
atoms	Wyckoff position	*x/a*	*y/b*	*z/c*	Occ.	B_{iso}
Al	4 a	0.037(2)	0.483(3)	0.206(3)	1	2.3(5)
Cl1	4 a	0.031(1)	0.490(3)	0.549(2)	1	3.0(2)
Cl2	4 a	0.147(2)	0.316(2)	0.111(3)	1	3.0(2)
Cl3	4 a	0.348(1)	0.026(2)	0.925(3)	1	3.0(2)
Cl4	4 a	0.379(2)	0.337(2)	0.577(3)	1	3.0(2)
Na	4 a	0.125(3)	0.219(1)	0.698(5)	1	6.5(9)

Table S3: Structural parameters obtained from Rietveld refinement of Na_2ZnCl_4

NZC						
S.G.: *Pnma* (62); Z = 4						R_{Bragg} = 3.93%
a = 13.7197(4) Å; *b* = 8.0715(3) Å; *c* = 6.4164(2) Å; *V* = 710.547(36) Å^3; *V/Z* = 177.637(9) Å^3						R_{wp} = 3.36%
						χ^2 = 5.99
		atomic position				
atoms	Wyckoff position	*x/a*	*y/b*	*z/c*	Occ.	B_{iso}
Zn	4 c	0.0928(3)	0.25	0.414(1)	1	2.3(1)
Cl1	4 c	0.0922(7)	0.25	0.763(1)	1	2.3(1)
Cl2	4 c	0.4394(7)	0.25	0.229(1)	1	2.3(1)
Cl3	8 d	0.1655(4)	0.027(1)	0.261(1)	2	2.3(1)
Na1	4 a	0	0	0	1	3.4(3)
Na2	4 c	0.2720(9)	0.25	-0.008(2)	1	3.4(3)

Table S4: Refined results of NAC and NZC phases of all $Na_{1+x}Al_{1-x}Zn_xCl_4$ samples.

(a)

Phase $NaAlCl_4$: $P2_12_12_1$ (19)

x	Unit cell parameters				Molar. fract. /%	Adding additional Zn in NAC phase	R_{Bragg}/%
	a/ Å	*b*/Å	*c*/ Å	*V*/Å^3			
0	10.349(1)	9.900(1)	6.177(1)	632.9(1)	100(0)	0	5.34
0.045	10.357(2)	9.902(2)	6.178(1)	632.6(2)	97.1(32)	-0.011(26)	5.68
0.06	10.363(2)	9.903(2)	6.180(1)	634.2(2)	96.7(34)	-0.011(23)	6.98
0.125	10.362(2)	9.902(2)	6.180(1)	634.2(2)	88.5(48)	0.005(37)	7.63
0.25	10.357(3)	9.900(3)	6.178(2)	633.5(4)	70.6(41)	-0.008(58)	5.66
0.4	10.360(5)	9.904(5)	6.180(3)	634.1(5)	46.2(49)	0.007(8)	6.36
0.5	10.349(6)	9.900(6)	6.186(3)	633.8(6)	27.7(20)	-0.053(90)	6.44

**x*: Nominal *x* in $Na_{1+x}Al_{1-x}Zn_xCl_4$

The additional Zn amount in the NAC phase equals the Na2 amount in the NAC phase (Occ_{Zn}=Occ_{Na2}).

(b)

Phase Na_2ZnCl_4: *Pnma* (62)

x	Unit cell parameters				Molar. fract. /%	Na1 Occ. In NZC phase	Na2 Occ. in NZC phase	Zn Occ. on Zn site	R_{Bragg}/%
	a/ Å	*b*/Å	*c*/ Å	*V*/Å^3					
0.045	13.680	8.039	6.410	704.942	2.9(4)	0.699	0.987	0.684	18.7
0.06	13.680	8.039	6.410	704.942	3.3(5)	0.699	0.987	0.684	13.9
0.125	13.680	8.039	6.410	704.942	11.5(14)	0.699	0.987	0.684	15.2
0.25	13.68(1)	8.039(7)	6.410(4)	704.94(10)	29.4(20)	0.699(131)	0.987(116)	0.684(87)	8.28
0.4	13.687(9)	8.039(5)	6.415(3)	705.83 (71)	53.8(41)	0.735(90)	0.932(90)	0.674(60)	5.75
0.5	13.704(3)	8.041(2)	6.414(1)	706.72(28)	72.3(41)	0.781(43)	0.933(48)	0.708(43)	4.58
0.625	13.701(3)	8.037(2)	6.408(1)	705.66(26)	100	0.736(35)	0.944(39)	0.673(31)	4.38
0.75	13.720(3)	8.044(2)	6.407(1)	707.06(27)	100	0.845(38)	0.907(38)	0.752(38)	6.08
0.95	13.716(4)	8.057(3)	6.406(2)	708.01(36)	100	0.948(61)	0.994(61)	0.941(61)	7.03
1	13.7197(4)	8.0715(3)	6.4164(2)	710.547(36)	100	1	1	1	3.93

**x*: Nominal *x* in $Na_{1+x}Al_{1-x}Zn_xCl_4$

The Zn site in the NZC phase is constrained to be fully occupied by Al and Zn (Occ_{Zn}+Occ_{Al}=1)

Table S5: Structural parameters obtained from Rietveld refinement of NZAC (x = 0.045).

**NAC (x = 0.045)**						
S.G.: $P\ 2_12_12_1$ (19); Z = 4						R_{Bragg} = 5.68%
a = 10.357(2) Å; b = 9.902(2) Å; c = 6.178(1) Å; V = 632.58(22) $Å^3$; V/Z = 158.145(55) $Å^3$						R_{wp} = 6.46%
Wt.Fract. (%) = 95.33(63)						χ^2 = 22.10
		atomic position				
atoms	Wyckoff position	*x/a*	*y/b*	*z/c*	Occ.	B_{iso}
Al	4 a	0.036(4)	0.477(5)	0.202(5)	1	1.4(9)
Cl1	4 a	0.032(3)	0.495(5)	0.551(4)	1	2.8(3)
Cl2	4 a	0.150(3)	0.317(4)	0.107(5)	1	2.8(3)
Cl3	4 a	0.347(2)	0.025(3)	0.926(5)	1	2.8(3)
Cl4	4 a	0.380(3)	0.335(3)	0.577(6)	1	2.8(3)
Na	4 a	0.121(6)	0.222(5)	0.701(9)	1	4.0

**NZC (x = 0.045)**						
S.G.: *Pnma* (62); Z = 4						R_{Bragg} = 18.7%
a = 13.680 Å; b = 8.039 Å; c = 6.410 Å; V = 704.942 $Å^3$; V/Z = 176.2355 $Å^3$						R_{wp} = 6.46%
Wt. Fract.(%) = 3.77(10)						χ^2 = 22.10
		atomic position				
atoms	Wyckoff position	*x/a*	*y/b*	*z/c*	Occ.	B_{iso}
Zn	4 c	0.09082	0.25	0.41635	0.68404	2.02926
Al	4 c	0.09082	0.25	0.41635	0.31596	2.02926
Cl1	4 c	0.087	0.25	0.75491	1	2.02926
Cl2	4 c	0.44012	0.25	0.22462	1	2.02926
Cl3	8 d	0.16113	0.0459	0.25837	2	2.02926
Na1	4 a	0	0	0	0.69932	2.02926
Na2	4 c	0.28021	0.25	0.00021	0.98745	2.02926

Table S6: Structural parameters obtained from Rietveld refinement of NZAC (*x* = 0.06).

**NAC (*x* = 0.06)**						
S.G.: *P* $2_12_12_1$ (19); Z = 4						R_{Bragg} = 6.98%
a = 10.363(2) Å; *b* = 9.903(2) Å; *c* = 6.180(1) Å; *V* = 634.20(18) Å^3; *V/Z* = 158.55(4) Å^3						R_{wp} = 6.84%
Wt. Fract. (%) = 93.09 (65)						χ^2 = 35.20
		atomic position				
atoms	Wyckoff position	*x/a*	*y/b*	*z/c*	Occ.	B_{iso}
Al	4 a	0.034(3)	0.477(4)	0.202(4)	1	0.3(6)
Cl1	4 a	0.031(2)	0.499(4)	0.550(3)	1	1.9(2)
Cl2	4 a	0.151(2)	0.318(3)	0.109(4)	1	1.9(2)
Cl3	4 a	0.348(2)	0.02719	0.929(4)	1	1.9(2)
Cl4	4 a	0.381(3)	0.332(2)	0.579(5)	1	1.9(2)
Na	4 a	0.120(4)	0.224(4)	0.697(7)	1	4.0

**NZC (*x* = 0.06)**						
S.G.: *Pnma* (62); Z = 4						R_{Bragg} = 13.9%
a = 13.680 Å; *b* = 8.039 Å; *c* = 6.410 Å; *V* = 704.942 Å^3; *V/Z* = 176.2355 Å^3						R_{wp} = 6.84%
Wt. Fract. (%) = 4.24 (12)						χ^2 = 35.20
		atomic position				
atoms	Wyckoff position	*x/a*	*y/b*	*z/c*	Occ.	B_{iso}
Zn	4 c	0.09082	0.25	0.41635	0.68404	2.02926
Al	4 c	0.09082	0.25	0.41635	0.31596	2.02926
Cl1	4 c	0.087	0.25	0.75491	1	2.02926
Cl2	4 c	0.44012	0.25	0.22462	1	2.02926
Cl3	8 d	0.16113	0.0459	0.25837	2	2.02926
Na1	4 a	0	0	0	0.69932	2.02926
Na2	4 c	0.28021	0.25	0.00021	0.98745	2.02926

Table S7: Structural parameters obtained from Rietveld refinement of NZAC (x = 0.125).

**NAC (x = 0.125)**						
S.G.: $P\,2_12_12_1$ (19); Z = 4						R_{Bragg} = 7.63%
a = 10.362(2) Å; b = 9.902(2) Å; c = 6.180(1) Å; V = 634.16(24) Å^3; V/Z = 158.54(6) Å^3						R_{wp} = 8.61%
Wt. Fract. (%) = 83.71(90)						χ^2 = 46.70
		atomic position				
atoms	Wyckoff position	*x/a*	*y/b*	*z/c*	Occ.	B_{iso}
Al	4 a	0.037(4)	0.475(6)	0.203(6)	1	0.8(10)
Cl1	4 a	0.034(3)	0.494(6)	0.552(5)	1	3.4(5)
Cl2	4 a	0.149(4)	0.319(5)	0.106(6)	1	3.4(5)
Cl3	4 a	0.345(3)	0.025(4)	0.925(7)	1	3.4(5)
Cl4	4 a	0.379(5)	0.334(4)	0.579(8)	1	3.4(5)
Na	4 a	0.121(7)	0.225(6)	0.71(1)	1	4.0

**NZC (x = 0.125)**						
S.G.: *Pnma* (62); Z = 4						R_{Bragg} = 15.2%
a = 13.680 Å; b = 8.039 Å; c = 6.410 Å; V = 704.942 Å^3; V/Z = 176.2355 Å^3						R_{wp} = 8.61%
Wt. Fract. (%) = 14.3(3)						χ^2 = 46.70
		atomic position				
atoms	Wyckoff position	*x/a*	*y/b*	*z/c*	Occ.	B_{iso}
Zn	4 c	0.09082	0.25	0.41635	0.68404	2.02926
Al	4 c	0.09082	0.25	0.41635	0.31596	2.02926
Cl1	4 c	0.087	0.25	0.75491	1	2.02926
Cl2	4 c	0.44012	0.25	0.22462	1	2.02926
Cl3	8 d	0.16113	0.0459	0.25837	2	2.02926
Na1	4 a	0	0	0	0.69932	2.02926
Na2	4 c	0.28021	0.25	0.00021	0.98745	2.02926

Table S8: Structural parameters obtained from Rietveld refinement of NZAC (x = 0.25).

**NAC (x = 0.25)**						
S.G.: $P\ 2_12_12_1$ (19); Z = 4						R_{Bragg} = 5.66%
a = 10.357(3) Å; b = 9.900(3) Å; c = 6.178(2) Å; V = 633.484(356) Å^3; V/Z = 158.371(89) Å^3						R_{wp} = 5.03%
Wt. Fract. (%) = 65.17(76)						χ^2 = 12.2
		atomic position				
atoms	Wyckoff position	*x/a*	*y/b*	*z/c*	Occ.	B_{iso}
Al	4 a	0.038(8)	0.48(1)	0.20(1)	1	3(2)
Cl1	4 a	0.031(5)	0.491(9)	0.555(8)	1	2.8(7)
Cl2	4 a	0.144(7)	0.315(7)	0.11(1)	1	2.8(7)
Cl3	4 a	0.349(5)	0.024(6)	0.93(1)	1	2.8(7)
Cl4	4 a	0.377(7)	0.336(6)	0.58(1)	1	2.8(7)
Na	4 a	0.12(1)	0.223(9)	0.71(2)	1	4.0

**NZC (x = 0.25)**						
S.G.: *Pnma* (62); Z = 4						R_{Bragg} = 8.28%
a = 13.68(1) Å; b = 8.039(7) Å; c = 6.410(4) Å; V = 704.94(102) Å^3; V/Z = 176.236(254) Å^3						R_{wp} = 5.03%
Wt. Fract. (%) = 33.11(45)						χ^2 = 12.2
		atomic position				
atoms	Wyckoff position	*x/a*	*y/b*	*z/c*	Occ.	B_{iso}
Zn	4 c	0.091(5)	0.25	0.416(9)	0.68(9)	2.0(6)
Al	4 c	0.091(5)	0.25	0.416(9)	0.32(9)	2.0(6)
Cl1	4 c	0.087(9)	0.25	0.75(1)	1	2.0(6)
Cl2	4 c	0.440(9)	0.25	0.22(1)	1	2.0(6)
Cl3	8 d	0.161(6)	0.0459	0.26(1)	2	2.0(6)
Na1	4 a	0	0	0	0.70(13)	2.0(6)
Na2	4 c	0.28(1)	0.25	0.00(3)	0.99(12)	2.0(6)

Table S9: Structural parameters obtained from Rietveld refinement of NZAC (x = 0.40).

**NAC (x = 0.40)**						
S.G.: $P\ 2_12_12_1$ (19); Z = 4						R_{Bragg} = 6.36%
a = 10.360(5) Å; b = 9.904(5) Å; c = 6.180(3) Å; V = 634.096(498) Å^3; V/Z = 158.524(124) Å^3						R_{wp} = 5.27%
Wt. Fract. (%) = 41.02(58)						χ^2 = 18.5
		atomic position				
atoms	Wyckoff position	*x/a*	*y/b*	*z/c*	Occ.	B_{iso}
Al	4 a	0.03(1)	0.47(1)	0.19(2)	1	2.5
Cl1	4 a	0.028(8)	0.51(1)	0.56(1)	1	1.3(9)
Cl2	4 a	0.154(9)	0.324(9)	0.10(1)	1	1.3(9)
Cl3	4 a	0.350(7)	0.027(8)	0.92(1)	1	1.3(9)
Cl4	4 a	0.387(8)	0.328(9)	0.59(2)	1	1.3(9)
Na	4 a	0.11(2)	0.23(2)	0.71(3)	1	4.0

**NZC (x = 0.40)**						
S.G.: *Pnma* (62); Z = 4						R_{Bragg} = 5.75%
a = 13.687(9) Å; b = 8.039(5) Å; c = 6.415(3) Å; V = 705.827(709) Å^3; V/Z = 176.457(177) Å^3						R_{wp} = 5.27%
Wt. Fract. (%) = 58.01(59)						χ^2 = 18.5
		atomic position				
atoms	Wyckoff position	*x/a*	*y/b*	*z/c*	Occ.	B_{iso}
Zn	4 c	0.092(3)	0.25	0.414(7)	0.67(6)	1.9(4)
Al	4 c	0.092(3)	0.25	0.414(7)	0.33(6)	1.9(4)
Cl1	4 c	0.095(6)	0.25	0.74(1)	1	1.9(4)
Cl2	4 c	0.442(6)	0.25	0.225(9)	1	1.9(4)
Cl3	8 d	0.165(4)	0.033(6)	0.247(9)	2	1.9(4)
Na1	4 a	0	0	0	0.73(9)	1.9(4)
Na2	4 c	0.275(7)	0.25	-0.01(2)	0.93(10)	1.9(4)

Table S10: Structural parameters obtained from Rietveld refinement of NZAC (x = 0.50).

**NAC (x = 0.50)**						
S.G.: $P\ 2_12_12_1$(19); Z = 4						R_{Bragg} = 6.44%
a = 10.349(6) Å; b = 9.900(6) Å; c = 6.186(3) Å; V = 633.768(575) Å^3; V/Z = 158.442(144) Å^3						R_{wp} = 5.49%
Wt. Fract. (%) = 23.20(36)						χ^2 = 91.9
		atomic position				
atoms	Wyckoff position	*x/a*	*y/b*	*z/c*	Occ.	B_{iso}
Al	4 a	0.04(1)	0.52(2)	0.24(2)	1	2.5
Cl1	4 a	0.035(9)	0.53(1)	0.63(2)	1	1.32
Cl2	4 a	0.15(1)	0.36(1)	0.12(2)	1	1.32
Cl3	4 a	0.315(7)	0.00(1)	0.89(2)	1	1.32
Cl4	4 a	0.34(1)	0.28(1)	0.63(2)	1	1.32
Na	4 a	0.10(2)	0.26(2)	0.77(3)	1	4

**NZC (x = 0.50)**						
S.G.: P*nma* (62); Z = 4						R_{Bragg} = 4.58%
a = 13.704(3) Å; b = 8.041(2) Å; c = 6.414(1) Å; V = 706.720(280) Å^3; V/Z = 176.68(7) Å^3						R_{wp} = 5.49%
Wt. Fract. (%) = 74.44(89)						χ^2 = 91.9
		atomic position				
atoms	Wyckoff position	*x/a*	*y/b*	*z/c*	Occ.	B_{iso}
Zn	4 c	0.091(1)	0.25	0.408(3)	0.71 (4)	1.0(4)
Al	4 c	0.091(1)	0.25	0.408(3)	0.29(4)	1.0(4)
Cl1	4 c	0.095(3)	0.25	0.745(5)	1	2.9(5)
Cl2	4 c	0.439(2)	0.25	0.228(4)	1	2.9(5)
Cl3	8 d	0.165(2)	0.034(3)	0.253(4)	2	2.9(5)
Na1	4 a	0	0	0	0.78(5)	3(1)
Na2	4 c	0.277(3)	0.25	-0.003(8)	0.93(5)	3(1)

Table S11: Structural parameters obtained from Rietveld refinement of NZAC (x = 0.625, phase pure) (a)before and (b)after post-annealing process.

**a) Pristine NZC (x = 0.625)**						
S.G.: *Pnma* (62); Z = 4						R_{Bragg} = 4.38 %
a = 13.701(3) Å; b = 8.037(2) Å; c = 6.408(1) Å; V = 705.656(257) Å^3; V/Z = 176.414(64) Å^3						R_{wp} = 4.75%
Wt. Fract. (%) = 100(1). crystallite size ~ 36nm						χ^2 = 10.6
		atomic position				
atoms	Wyckoff position	*x/a*	*y/b*	*z/c*	Occ.	B_{iso}
Zn	4 c	0.093(1)	0.25	0.413(2)	0.67(3)	0.4(3)
Al	4 c	0.093(1)	0.25	0.413(2)	0.33(3)	0.4(3)
Cl1	4 c	0.091(2)	0.25	0.753(3)	1	3.1(4)
Cl2	4 c	0.441(2)	0.25	0.225(3)	1	3.1(4)
Cl3	8 d	0.166(2)	0.034(2)	0.253(3)	2	3.1(4)
Na1	4 a	0	0	0	0.74(4)	1.1(7)
Na2	4 c	0.277(2)	0.25	-0.008(6)	0.94(4)	1.1(7)

**b) Post-annealed NZC (x = 0.625)**						
S.G.: P*nma* (62); Z = 4						R_{Bragg} = 5.95%
a = 13.694(3) Å; b = 8.044(2) Å; c = 6.419(1) Å; V = 707.126(267) Å^3; V/Z = 176.782(67) Å^3						R_{wp} = 5.14%
Wt. Fract. (%) = 100(1). crystallite size ~ 50nm						χ^2 = 16.4
		atomic position				
atoms	Wyckoff position	*x/a*	*y/b*	*z/c*	Occ.	B_{iso}
Zn	4 c	0.091(1)	0.25	0.409(2)	0.67(3)	2.0(3)
Al	4 c	0.091(1)	0.25	0.409(2)	0.32(3)	2.0(3)
Cl1	4 c	0.094(2)	0.25	0.754(4)	1	3.9(4)
Cl2	4 c	0.439(2)	0.25	0.221(3)	1	3.9(4)
Cl3	8 d	0.165(2)	0.030(2)	0.247(3)	2	3.9(4)
Na1	4 a	0	0	0	0.72(3)	3.3(8)
Na2	4 c	0.277(3)	0.25	-0.009(7)	0.96(3)	3.3(8)

Table S12. Structural parameters obtained from Rietveld refinement of NZAC (x = 0.75).

NZC (x = 0.75)						
S.G.: *Pnma* (62); Z = 4						R_{Bragg} = 6.08%
a = 13.720(3) Å; b = 8.044(2) Å; c = 6.407(1) Å; V = 707.064(273)Å^3; V/Z = 176.766(68) Å^3						R_{wp} = 4.50%
						χ^2 = 7.78
		atomic position				
atoms	Wyckoff position	*x/a*	*y/b*	*z/c*	Occ.	B_{iso}
Zn	4 c	0.092(1)	0.25	0.411(2)	0.75(4)	0.2(3)
Al	4 c	0.092(1)	0.25	0.411(2)	0.25(4)	0.2(3)
Cl1	4 c	0.094(3)	0.25	0.756(4)	1	3.4(5)
Cl2	4 c	0.439(3)	0.25	0.230(4)	1	3.4(5)
Cl3	8 d	0.167(2)	0.029(2)	0.261(4)	2	3.4(5)
Na1	4 a	0	0	0	0.85(4)	2.0(8)
Na2	4 c	0.275(3)	0.25	-0.013(7)	0.91(4)	2.0(8)

Table S13. Structural parameters obtained from Rietveld refinement of NZAC (x = 0.95).

NZC (x = 0.95)						
S.G.: *Pnma* (62); Z = 4						R_{Bragg} = 7.03%
a = 13.716(4) Å; b = 8.057(3) Å; c = 6.406(2) Å; V = 708.012(357) Å^3; V/Z = 177.003(89) Å^3						R_{wp} = 5.95%
						χ^2 = 12.0
		atomic position				
atoms	Wyckoff position	*x/a*	*y/b*	*z/c*	Occ.	B_{iso}
Zn	4 c	0.092(2)	0.25	0.413(3)	0.94(7)	0.9(4)
Al	4 c	0.092(2)	0.25	0.413(3)	0.06(7)	0.9(4)
Cl1	4 c	0.091(3)	0.25	0.760(5)	1	2.3(7)
Cl2	4 c	0.439(3)	0.25	0.231(5)	1	2.3(7)
Cl3	8 d	0.165(2)	0.030(3)	0.262(5)	2	2.3(7)
Na1	4 a	0	0	0	0.95(6)	2(1)
Na2	4 c	0.272(4)	0.25	-0.012(9)	0.99(7)	2(1)

Performance of MACE-MP-0/D3(BJ)

Table S14 compares the investigated structures' energies and forces obtained with PBE/D3(BJ) and MACE-MP-0/D3(BJ).

Table S14. Deviation of energies and forces for Na_2ZnCl_4, $Na_{1.75}Zn_{0.75}Al_{0.25}Cl_4$ A and B, and $Na_{1.625}Zn_{0.625}Al_{0.375}Cl_4$.

	Δ Energy	RMSD Force
Structure	meV/atom	meV/Å
Na_2ZnCl_4	-16.6	22.4
$Na_{1.75}Zn_{0.75}Al_{0.25}Cl_4$ **A**	-13.0	25.7
$Na_{1.75}Zn_{0.75}Al_{0.25}Cl_4$ **B**	-13.9	32.9
$Na_{1.625}Zn_{0.625}Al_{0.375}Cl_4$	-15.8	64.7

MACE-MP-0/D3(BJ) generally underestimates the energies of the systems compared to PBE/D3(BJ). The forces for Na_2ZnCl_4 and $Na_{1.75}Zn_{0.75}Al_{0.25}Cl_4$ are in a sensible range of < 35 meV/Å while the forces for $Na_{1.625}Zn_{0.625}Al_{0.375}Cl_4$ have a significantly larger error of 65 meV/Å. This would still be within the acceptable range given by VASP for their machine-learned potential [33] and it matches the performance of other machine-learned potentials.[34]

Table S15. Lattice Parameters for Na_2ZnCl_4, $Na_{1.75}Zn_{0.75}Al_{0.25}Cl_4$ and $Na_{1.625}Zn_{0.625}Al_{0.375}Cl_4$ with PBE/D3(BJ) and MACE-MP-0/D3(BJ).

	PBE/D3(BJ)			MACE-MP-0/D3(BJ)			Deviation (%)
Structure	a	b	c	a	b	C	
Na_2ZnCl_4	13.52	7.97	6.39	13.54	7.96	6.43	0.22 ± 0.38
$Na_{1.75}Zn_{0.75}Al_{0.25}Cl_4$ **A**	26.64	7.93	6.39	26.74	7.93	6.42	0.28 ± 0.24
$Na_{1.75}Zn_{0.75}Al_{0.25}Cl_4$ **B**	26.51	8.04	6.37	26.51	8.00	6.40	0.00 ± 0.54
$Na_{1.625}Zn_{0.625}Al_{0.375}Cl_4$	26.89	7.99	6.38	26.45	7.97	6.42	-0.42 ±0.56

The lattice parameters obtained with MACE-MP-0/D3(BJ) match the PBE/D3(BJ) results within less than 1 % and the bond lengths for the Al-Cl, Zn-Cl, and Na-Cl bonds are within less than 0.5 %. The structures are reproduced very well.

Table S16. Extracted NMR parameters from the 2D ^{23}Na MQMAS NMR spectra.

Site	δ_{iso} [ppm]	C_Q [MHz]	η
**Na1 (NZC)**	4.8	0.67	0.17
**Na2 (NZC)**	0.4	0.5	0.0
**Na1 (Zn/Al) 1**	2.3	1.1	0.5
**Na2 (Zn/Al) 2**	-1.7	0.4	0.0
**Na2 (Zn/Al) 3**	-6.6	0.3	0.0
**Na2 (Zn/Al) 4**	-8.6	0.5	0.0
**Na1 (NAC)**	-14.1	1.04	0.05
**NaCl**	7.0	0.0	-

Table S17. Parameters for the deconvolution of the 1D ^{23}Na MAS NMR spectrum for the *x* = 0.75 composition are shown in Figure 10a.

Assignment	Isotropic chemical shift δiso	Quadrupolar coupling CQ	Asymmetry η	Integral	Relative integral	Lorentz boadening [Hz]	Gauss broadening [ppm]
**NaCl**	7.0	0.0	0.00	1.84E+10	~	98.6	2.49E-08
**Na1**	4.8	0.7	0.17	6.70E+10	0.31	120.1	1.301
**Na1 (Zn/Al)**	2.3	1.1	0.50	4.34E+10	0.20	0.7	3.510
**Na2**	0.4	0.5	0.025	6.33E+10	0.29	51.3	1.254
**Na2 (Zn/Al)**	-1.7	0.4	0.00	1.74E+10	0.08	35.3	3.645
**Na2 (Zn/Al)**	-6.6	0.3	0.00	2.80E+10	0.13	138.8	4.652
**Na2 (Zn/Al)**	-8.6	0.5	0.00	0	0.00	121.8	5.800

Table S18. Parameters for the deconvolution of the 1D ^{23}Na MAS NMR spectrum for the *x* = 0.5 composition are shown in Figure 10c.

Assignment	Isotropic chemical shift δ_{iso}	Quadrupolar coupling C_Q	Asymmetry η	Integral	Relative integral	Lorentz boadening [Hz]	Gauss broadening [ppm]
**NaCl**	7.0	0.0	0.00	1.75E+10	~	109.2	0.0
**Na1**	4.8	0.7	0.17	4.56E+10	0.22	106.8	1.22
**Na1 (Zn/Al)**	2.3	1.1	0.50	4.09E+10	0.20	0.7	3.51
**Na2**	0.4	0.5	0.025	4.02E+10	0.19	45.8	1.36
**Na2 (Zn/Al)**	-1.7	0.4	0.00	1.91E+10	0.09	35.3	3.65
**Na2 (Zn/Al)**	-6.6	0.3	0.00	3.47E+10	0.17	138.8	4.65
**Na2 (Zn/Al)**	-8.6	0.5	0.00	1.41E+10	0.07	121.8	5.80
**Na1 in NAC**	-14.2	1.1	0.05	1.25E+10	0.06	77.9	0.14

Table S19. Summary of calculated NMR parameters for the different models. For referencing the absolute chemical shielding, a linear relation was used (δ_{ref}=1.27*σ_{abs}+542.99), which was derived from fitting a linear function to experimentally observed isotropic chemical shifts δ_{iso} of Na_2ZnCl_4 and $NaAlCl_4$ vs. the calculated absolute chemical shielding.

Model	Type of site	No of surr. Al	No of surr. Zn	Absolute chemical shielding	Referenced chemical shift [ppm]	Absolute value of quadrupolar coupling [MHz]	Asymmetry
**Na_2ZnCl_4**	Na1	0	4	-537.163	4.59	0.70	0.58
**Na_2ZnCl_4**	Na2	0	5	-542.082	0.72	0.61	0.25
**$NaAlCl_4$**	Na1		0	-561.102	-14.26	1.32	0.25
**$Na_{1.75}Zn_{0.75}Al_{0.25}Cl_4$ w. Na1 vac.**	Na1	0	4	-537.526	4.30	0.68	0.85
**$Na_{1.75}Zn_{0.75}Al_{0.25}Cl_4$ w. Na1 vac.**	Na1	0	4	-536.967	4.74	0.68	0.63

**$Na_{1.75}Zn_{0.75}Al_{0.25}Cl_4$ w. Na1 vac.**	Na1	0	4	-536.967	4.74	0.68	0.63
**$Na_{1.75}Zn_{0.75}Al_{0.25}Cl_4$ w. Na1 vac.**	Na1	0	4	-537.526	4.30	0.67	0.85
**$Na_{1.75}Zn_{0.75}Al_{0.25}Cl_4$ w. Na1 vac.**	Na1	0	4	-537.526	4.30	0.67	0.85
**$Na_{1.75}Zn_{0.75}Al_{0.25}Cl_4$ w. Na1 vac.**	Na1	0	4	-537.526	4.30	0.67	0.85
**$Na_{1.75}Zn_{0.75}Al_{0.25}Cl_4$ w. Na1 vac.**	Na2	0	5	-542.596	0.31	0.56	0.23
**$Na_{1.75}Zn_{0.75}Al_{0.25}Cl_4$ w. Na1 vac.**	Na2	0	5	-542.091	0.71	0.59	0.08
**$Na_{1.75}Zn_{0.75}Al_{0.25}Cl_4$ w. Na1 vac.**	Na2	0	5	-542.091	0.71	0.59	0.08
**$Na_{1.75}Zn_{0.75}Al_{0.25}Cl_4$ w. Na1 vac.**	Na2	3	5	-549.917	-5.45	0.69	0.19
**$Na_{1.75}Zn_{0.75}Al_{0.25}Cl_4$ w. Na1 vac.**	Na2	3	5	-549.917	-5.45	0.69	0.19
**$Na_{1.75}Zn_{0.75}Al_{0.25}Cl_4$ w. Na1 vac.**	Na2	2	3	-545.096	-1.66	0.45	0.96
**$Na_{1.75}Zn_{0.75}Al_{0.25}Cl_4$ w. Na1 vac.**	Na2	2	3	-545.096	-1.66	0.45	0.96
**$Na_{1.75}Zn_{0.75}Al_{0.25}Cl_4$ w. Na1 vac.**	Na2	0	5	-542.596	0.31	0.56	0.23
**$Na_{1.75}Zn_{0.75}Al_{0.25}Cl_4$ w. Na2 vac.**	Na1	0	4	-537.846	4.10	0.66	0.68
**$Na_{1.75}Zn_{0.75}Al_{0.25}Cl_4$ w. Na2 vac**	Na1	0	4	-537.846	4.10	0.66	0.68
**$Na_{1.75}Zn_{0.75}Al_{0.25}Cl_4$ w. Na2 vac**	Na1	0	4	-537.846	4.10	0.66	0.68
**$Na_{1.75}Zn_{0.75}Al_{0.25}Cl_4$ w. Na2 vac**	Na1	0	4	-537.846	4.10	0.66	0.68
**$Na_{1.75}Zn_{0.75}Al_{0.25}Cl_4$ w. Na2 vac**	Na1	2	2	-541.531	1.24	1.09	0.58
**$Na_{1.75}Zn_{0.75}Al_{0.25}Cl_4$ w. Na2 vac**	Na1	2	2	-541.531	1.24	1.09	0.58

**$Na_{1.75}Zn_{0.75}Al_{0.25}Cl_4$ w. Na2 vac**	Na1	2	2	-541.531	1.24	1.09	0.58
**$Na_{1.75}Zn_{0.75}Al_{0.25}Cl_4$ w. Na2 vac**	Na1	2	2	-541.531	1.24	1.09	0.58
**$Na_{1.75}Zn_{0.75}Al_{0.25}Cl_4$ w. Na2 vac**	Na2	0	5	-542.581	0.43	0.76	0.05
**$Na_{1.75}Zn_{0.75}Al_{0.25}Cl_4$ w. Na2 vac**	Na2	0	5	-542.581	0.43	0.76	0.05
**$Na_{1.75}Zn_{0.75}Al_{0.25}Cl_4$ w. Na2 vac**	Na2	1	4	-543.548	-0.32	0.81	0.73
**$Na_{1.75}Zn_{0.75}Al_{0.25}Cl_4$ w. Na2 vac**	Na2	1	4	-543.548	-0.32	0.81	0.73
**$Na_{1.75}Zn_{0.75}Al_{0.25}Cl_4$ w. Na2 vac**	Na2	0	5	-542.349	0.61	0.81	0.36
**$Na_{1.75}Zn_{0.75}Al_{0.25}Cl_4$ w. Na2 vac**	Na2	0	5	-542.349	0.61	0.81	0.36

References

(1) Amoureux, J.-P.; Fernandez, C.; Steuernagel, S. *Z*Filtering in MQMAS NMR. *Journal of Magnetic Resonance, Series A* **1996**, *123* (1), 116–118. https://doi.org/10.1006/jmra.1996.0221.

(2) Van Meerten, S. G. J.; Franssen, W. M. J.; Kentgens, A. P. M. ssNake: A Cross-Platform Open-Source NMR Data Processing and Fitting Application. *Journal of Magnetic Resonance* **2019**, *301*, 56–66. https://doi.org/10.1016/j.jmr.2019.02.006.

(3) Kresse, G.; Hafner, J. Ab Initio Molecular Dynamics for Liquid Metals. *Phys. Rev. B* **1993**, *47* (1), 558–561. https://doi.org/10.1103/PhysRevB.47.558.

(4) Kresse, G.; Furthmüller, J. Efficiency of Ab-Initio Total Energy Calculations for Metals and Semiconductors Using a Plane-Wave Basis Set. *Computational Materials Science* **1996**, *6* (1), 15–50. https://doi.org/10.1016/0927-0256(96)00008-0.

(5) Kresse, G.; Furthmüller, J. Efficient Iterative Schemes for Ab Initio Total-Energy Calculations Using a Plane-Wave Basis Set. *Phys. Rev. B* **1996**, *54* (16), 11169–11186. https://doi.org/10.1103/PhysRevB.54.11169.

(6) Kresse, G.; Joubert, D. From Ultrasoft Pseudopotentials to the Projector Augmented-Wave Method. *Phys. Rev. B* **1999**, *59* (3), 1758–1775. https://doi.org/10.1103/PhysRevB.59.1758.

(7) Perdew, J. P.; Burke, K.; Ernzerhof, M. Generalized Gradient Approximation Made Simple. *Phys. Rev. Lett.* **1996**, *77* (18), 3865–3868. https://doi.org/10.1103/PhysRevLett.77.3865.

(8) Jain, A.; Hautier, G.; Moore, C. J.; Ping Ong, S.; Fischer, C. C.; Mueller, T.; Persson, K. A.; Ceder, G. A High-Throughput Infrastructure for Density Functional Theory Calculations. *Computational Materials Science* **2011**, *50* (8), 2295–2310. https://doi.org/10.1016/j.commatsci.2011.02.023.

(9) Grimme, S.; Ehrlich, S.; Goerigk, L. Effect of the Damping Function in Dispersion Corrected Density Functional Theory. *Journal of Computational Chemistry* **2011**, *32* (7), 1456–1465. https://doi.org/10.1002/jcc.21759.

(10) Grimme, S.; Antony, J.; Ehrlich, S.; Krieg, H. A Consistent and Accurate Ab Initio Parametrization of Density Functional Dispersion Correction (DFT-D) for the 94 Elements H-Pu. *The Journal of Chemical Physics* **2010**, *132* (15), 154104. https://doi.org/10.1063/1.3382344.

(11) Ong, S. P.; Richards, W. D.; Jain, A.; Hautier, G.; Kocher, M.; Cholia, S.; Gunter, D.; Chevrier, V. L.; Persson, K. A.; Ceder, G. Python Materials Genomics (Pymatgen): A Robust, Open-Source Python Library for Materials Analysis. *Computational Materials Science* **2013**, *68*, 314–319. https://doi.org/10.1016/j.commatsci.2012.10.028.

(12) Larsen, A. H.; Mortensen, J. J.; Blomqvist, J.; Castelli, I. E.; Christensen, R.; Dułak, M.; Friis, J.; Groves, M. N.; Hammer, B.; Hargus, C.; Hermes, E. D.; Jennings, P. C.; Jensen, P. B.; Kermode, J.; Kitchin, J. R.; Kolsbjerg, E. L.; Kubal, J.; Kaasbjerg, K.; Lysgaard, S.; Maronsson, J. B.; Maxson, T.; Olsen, T.; Pastewka, L.; Peterson, A.; Rostgaard, C.; Schiøtz, J.; Schütt, O.; Strange, M.; Thygesen, K. S.; Vegge, T.; Vilhelmsen, L.; Walter, M.; Zeng, Z.; Jacobsen, K. W. The Atomic Simulation Environment—a Python Library for Working with Atoms. *J. Phys.: Condens. Matter* **2017**, *29* (27), 273002. https://doi.org/10.1088/1361-648X/aa680e.

(13) Batatia, I.; Benner, P.; Chiang, Y.; Elena, A. M.; Kovács, D. P.; Riebesell, J.; Advincula, X. R.; Asta, M.; Avaylon, M.; Baldwin, W. J.; Berger, F.; Bernstein, N.; Bhowmik, A.; Blau, S. M.; Cărare, V.; Darby, J. P.; De, S.; Pia, F. D.; Deringer, V. L.; Elijošius, R.; El-Machachi, Z.; Falcioni, F.; Fako, E.; Ferrari, A. C.; Genreith-Schriever, A.; George, J.; Goodall, R. E. A.; Grey, C. P.; Grigorev, P.; Han, S.; Handley, W.; Heenen, H. H.; Hermansson, K.; Holm, C.; Jaafar, J.; Hofmann, S.; Jakob, K. S.; Jung, H.; Kapil, V.; Kaplan, A. D.; Karimitari, N.; Kermode, J. R.; Kroupa, N.; Kullgren, J.; Kuner, M. C.; Kuryla, D.; Liepuoniute, G.; Margraf, J. T.; Magdău, I.-B.; Michaelides, A.; Moore, J. H.; Naik, A. A.; Niblett, S. P.; Norwood, S. W.; O'Neill, N.; Ortner, C.; Persson, K. A.; Reuter, K.; Rosen, A. S.; Schaaf, L. L.; Schran, C.; Shi, B. X.; Sivonxay, E.; Stenczel, T. K.; Svahn, V.; Sutton, C.; Swinburne, T. D.; Tilly, J.; Oord, C. van der; Varga-Umbrich, E.; Vegge, T.; Vondrák, M.; Wang, Y.; Witt, W. C.; Zills, F.; Csányi, G. A Foundation Model for Atomistic Materials Chemistry. arXiv March 1, 2024. https://doi.org/10.48550/arXiv.2401.00096.

(14) Batatia, I.; Kovacs, D. P.; Simm, G. N. C.; Ortner, C.; Csanyi, G. MACE: Higher Order Equivariant Message Passing Neural Networks for Fast and Accurate Force Fields; 2022.
(15) Batatia, I.; Batzner, S.; Kovacs, D.; Musaelian, A.; Simm, G.; Drautz, R.; Ortner, C.; Kozinsky, B.; Csányi, G. *The Design Space of E(3)-Equivariant Atom-Centered Interatomic Potentials*; 2022. https://doi.org/10.48550/arXiv.2205.06643.
(16) Takamoto, S.; Shinagawa, C.; Motoki, D.; Nakago, K.; Li, W.; Kurata, I.; Watanabe, T.; Yayama, Y.; Iriguchi, H.; Asano, Y.; Onodera, T.; Ishii, T.; Kudo, T.; Ono, H.; Sawada, R.; Ishitani, R.; Ong, M.; Yamaguchi, T.; Kataoka, T.; Hayashi, A.; Charoenphakdee, N.; Ibuka, T. Towards Universal Neural Network Potential for Material Discovery Applicable to Arbitrary Combination of 45 Elements. *Nat Commun* **2022**, *13* (1), 2991. https://doi.org/10.1038/s41467-022-30687-9.
(17) Okhotnikov, K.; Charpentier, T.; Cadars, S. Supercell Program: A Combinatorial Structure-Generation Approach for the Local-Level Modeling of Atomic Substitutions and Partial Occupancies in Crystals. *J Cheminform* **2016**, *8* (1), 17. https://doi.org/10.1186/s13321-016-0129-3.
(18) Melchionna, S.; Ciccotti, G.; Lee Holian, B. Hoover NPT Dynamics for Systems Varying in Shape and Size. *Molecular Physics* **1993**, *78* (3), 533–544. https://doi.org/10.1080/00268979300100371.
(19) Melchionna, S. Constrained Systems and Statistical Distribution. *Phys. Rev. E* **2000**, *61* (6), 6165–6170. https://doi.org/10.1103/PhysRevE.61.6165.
(20) Holian, B. L.; De Groot, A. J.; Hoover, W. G.; Hoover, C. G. Time-Reversible Equilibrium and Nonequilibrium Isothermal-Isobaric Simulations with Centered-Difference Stoermer Algorithms. *Phys. Rev. A* **1990**, *41* (8), 4552–4553. https://doi.org/10.1103/PhysRevA.41.4552.
(21) Henkelman, G.; Jónsson, H. Improved Tangent Estimate in the Nudged Elastic Band Method for Finding Minimum Energy Paths and Saddle Points. *The Journal of Chemical Physics* **2000**, *113* (22), 9978–9985. https://doi.org/10.1063/1.1323224.
(22) Henkelman, G.; Uberuaga, B. P.; Jónsson, H. A Climbing Image Nudged Elastic Band Method for Finding Saddle Points and Minimum Energy Paths. *The Journal of Chemical Physics* **2000**, *113* (22), 9901–9904. https://doi.org/10.1063/1.1329672.
(23) *Nudged elastic band method for finding minimum energy paths of transitions | Classical and Quantum Dynamics in Condensed Phase Simulations*. https://www.worldscientific.com/doi/abs/10.1142/9789812839664_0016 (accessed 2024-11-03).
(24) Sheppard, D.; Xiao, P.; Chemelewski, W.; Johnson, D. D.; Henkelman, G. A Generalized Solid-State Nudged Elastic Band Method. *The Journal of Chemical Physics* **2012**, *136* (7), 074103. https://doi.org/10.1063/1.3684549.
(25) *Paths to which the nudged elastic band converges - Sheppard - 2011 - Journal of Computational Chemistry - Wiley Online Library*. https://onlinelibrary.wiley.com/doi/10.1002/jcc.21748 (accessed 2024-11-03).
(26) Sheppard, D.; Terrell, R.; Henkelman, G. Optimization Methods for Finding Minimum Energy Paths. *The Journal of Chemical Physics* **2008**, *128* (13), 134106. https://doi.org/10.1063/1.2841941.
(27) Smidstrup, S.; Pedersen, A.; Stokbro, K.; Jónsson, H. Improved Initial Guess for Minimum Energy Path Calculations. *The Journal of Chemical Physics* **2014**, *140* (21), 214106. https://doi.org/10.1063/1.4878664.
(28) Lindgren, P.; Kastlunger, G.; Peterson, A. A. Scaled and Dynamic Optimizations of Nudged Elastic Bands. *J. Chem. Theory Comput.* **2019**, *15* (11), 5787–5793. https://doi.org/10.1021/acs.jctc.9b00633.
(29) Pyykkö, P. Year-2008 Nuclear Quadrupole Moments. *Molecular Physics* **2008**, *106* (16–18), 1965–1974. https://doi.org/10.1080/00268970802018367.

(30) Zagorac, D.; Müller, H.; Ruehl, S.; Zagorac, J.; Rehme, S. Recent Developments in the Inorganic Crystal Structure Database: Theoretical Crystal Structure Data and Related Features. *J Appl Cryst* **2019**, *52* (5), 918–925. https://doi.org/10.1107/S160057671900997X.
(31) Momma, K.; Izumi, F. VESTA 3 for Three-Dimensional Visualization of Crystal, Volumetric and Morphology Data. *J Appl Cryst* **2011**, *44* (6), 1272–1276. https://doi.org/10.1107/S0021889811038970.
(32) Hunter, J. D. Matplotlib: A 2D Graphics Environment. *Computing in Science & Engineering* **2007**, *9* (3), 90–95. https://doi.org/10.1109/MCSE.2007.55.
(33) *Understanding Molecular Simulation*; Elsevier, 2002. https://doi.org/10.1016/B978-0-12-267351-1.X5000-7.
(34) Röcken, S.; Zavadlav, J. Accurate Machine Learning Force Fields via Experimental and Simulation Data Fusion. *npj Comput Mater* **2024**, *10* (1), 1–10. https://doi.org/10.1038/s41524-024-01251-4.
